# Development of a behavioural welfare assessment tool for routine use with captive elephants

**DOI:** 10.1371/journal.pone.0210783

**Published:** 2019-02-06

**Authors:** Lisa Yon, Ellen Williams, Naomi D. Harvey, Lucy Asher

**Affiliations:** 1 School of Veterinary Medicine and Science, Faculty of Medical & Health Sciences, The University of Nottingham, Sutton Bonington, Leicestershire, United Kingdom; 2 School of Animal Rural and Environmental Sciences, Nottingham Trent University, Brackenhurst Campus, Southwell, Nottinghamshire, United Kingdom; 3 Centre for Behaviour and Evolution, Institute of Neuroscience, Newcastle University, Framlington Place, Newcastle, United Kingdom; University of Tasmania, AUSTRALIA

## Abstract

There has been much concern in recent years about the welfare of elephants in zoos across North America and Europe. While some previous studies have assessed captive elephant welfare at a particular point in time, there has been little work to develop methods which could be used for regular, routine welfare assessment. Such assessment is important in order to track changes in welfare over time. A welfare assessment tool should be rapid, reliable, and simple to complete, without requiring specialist training and facilities; welfare assessments based on behavioural observations are well suited to this purpose. This report describes the development of a new elephant behavioural welfare assessment tool designed for routine use by elephant keepers. Tool development involved: (i) identification of behavioural indicators of welfare from the literature and from focus groups with relevant stakeholders; (ii) development of a prototype tool; (iii) testing of the tool at five UK zoological institutions, involving 29 elephants (representing 46% of the total UK captive elephant population of 63 animals); (iv) assessment of feasibility and reliability of aspects of the prototype tool; (v) assessment of the validity of each element of the tool to reflect the relevant behaviour by comparing detailed behavioural observations with data from the prototype tool; (vi) assessment of known-groups criterion validity by comparing prototype tool scores in individuals with demographics associated with better or worse welfare; (vii) development of a finalised tool which incorporated all elements of the tool which met the criteria set for validity and reliability. Elements of the tool requiring further consideration are discussed, as are considerations for appropriate application and interpretation of scores. This novel behavioural welfare assessment tool can be used by elephant-holding facilities for routine behavioural welfare monitoring, which can inform adjustments to individual welfare plans for each elephant in their collection, to help facilities further assess and improve captive elephant welfare. This study provides an example of how an evidence-based behavioural welfare assessment tool for use by animal caretakers can be developed within the constraints of zoo-based research, which could be applied to a range of captive species.

## Introduction

Modern welfare assessment has placed much focus on providing animal carers or inspectors with the tools to be able to routinely assess welfare *in situ* (e.g. on farm, in the laboratory, in the field, in a rescue shelter and in zoos [1–4]). Routine assessment of welfare may be of particular importance for captive elephants. Zoo elephant welfare across North America and Europe has been criticised [5–9] and in the UK, specific concerns were raised by a report on the welfare of elephants in UK zoos [10]. A review of this report by the government advisory committee, the Zoos Forum [11], suggested that evidence of welfare improvements were needed in order for zoos to continue keeping elephants in captivity. Previous studies have focussed on judging the current welfare state of elephants [5, 10], but few studies have developed methods for routine assessment of elephant welfare. Yet, objective and regular assessment of elephant welfare is needed to be able to monitor and provide evidence of any improvements, as was mandated by the Zoos Forum and the House of Lords [11, 12].

Routine welfare assessment often needs to be rapid, non-invasive and should not require any specialist equipment, facilities or specific training of animals. For this reason routine welfare assessment is often based on observations of behaviour [13–15]. Measuring welfare is challenging even without such constraints, there is no single accepted welfare measure; multiple indicators of welfare should be used to surmise if an animal is in a good or bad welfare state [16]. Welfare indicators can nevertheless be objectively evaluated, according to how consistently they can be assessed (reliability), and according to level of evidence that the measurements reflect the construct they were designed to measure (validity). Indicators should differ between animals with better and worse welfare, should be repeatable, and the time frame of change should be known. A fully validated welfare tool will have assessed each type of validity and reliability (see Table 1) against predefined thresholds [17, 18] typically across multiple studies.

**Table 1:** Summary of the main types of reliability and validity applied to welfare assessment.

Reliability or Validity	Type of reliability or validity	Brief description
**Reliability**	Intra-rater reliability	Assess consistency when one person repeat-scores the animal within a short time period such as 2 to 7 days or ideally at the same time point
	Inter-rater reliability	Assess consistency when scorers simultaneously score the same animal within a short time period such as 2 to 7 days or ideally at the same time point.
	Test-re-test reliability	Assess consistency in scoring over a longer period (e.g. more than 2 weeks)
	Internal consistency	Assess the level of associations between grouped questions or measures
**Validity**	Content validity (e.g. face validity)	Assess whether individual questions really ask what they are meant to be asking
	Concurrent criterion validity	Compare measure to an independent “gold standard” measure
	Predictive or known groups criterion validity	Assess measures ability to predict a future outcome or distinguish between groups

There are a number of behavioural welfare indicators that might be used to assess the welfare of zoo elephants[19]. Stereotypies are one of those most frequently used [20]. Stereotypies are defined as ‘repetitive, invariant behaviour patterns with no obvious goal or function’ [21] and it is believed they are a way of coping with stress; however, the use of stereotypies as an indicator of current welfare state must be treated with caution, as there is evidence they can persist even after the stressor which caused their development is no longer present, so they may reflect a historical rather than current welfare state [22]. Veasey [23] suggested that documentation of baseline time budgets and comparison with time budgets in new environmental or social conditions, or comparison with wild elephant time budgets may be a valid means of measuring captive elephant welfare. Qualitative behavioural assessment (QBA) has been designed to capture information on the quality of an animal's demeanour. It has been shown to be useful for routine domestic animal welfare assessments [4, 24–28], and has been used to assess welfare in free-living African elephants [29]. Furthermore, demeanour was identified by elephant stakeholders as a potential welfare indicator [30]. Night-time and resting behaviour may also be a useful welfare indicator [31, 32]. Wild and captive elephants are known to spend much of the night active [33–38]. Many captive elephants do not have access to their outdoor enclosure at night, and are confined to their smaller indoor enclosures [10, 39], particularly during winter months in colder climates. Furthermore, keepers are usually not present during the night time to monitor behaviour; as this unmonitored time period often comprises more than half of each 24 hour period, it might be particularly important to measure welfare during the hours when keepers are not present. All UK elephant-holding zoos now have indoor video cameras to collect footage of their elephants overnight [40], but footage needs to be reviewed and assessed in order to monitor behaviour during this time.

The objective of this study was to develop a routine behavioural welfare assessment tool for keeper assessment of captive safari park and zoo elephants in the UK. Specifically, the aims were to develop and trial a prototype welfare assessment tool for elephants, to assess the reliability of the tool completed at multiple time points by multiple raters, and to assess validity of behavioural indicators in the tool, by comparing each to a more in-depth, objective behavioural assessment measuring the same behavioural welfare indicators. A final aim was to perform a known-groups validation by comparing scores from the tool in individuals with/without health conditions and with/without demographics associated with poor welfare in other studies. This work was undertaken as part of the activities of the British and Irish Association of Zoos and Aquariums (BIAZA) Elephant Welfare Group (see: https://biaza.org.uk/elephant-welfare-group).

## Methods

### Animals and housing

A prototype tool was tested at five elephant-holding facilities in the UK. These were selected to represent a range of facilities, including safari parks and zoos; different contact systems (free contact and protected contact); group sizes (4, 4, 5, 7 and 9); and levels of herd relatedness. In total the sample comprised 29 elephants (6 male, 23 female): 9 African (*Loxodonta Africana*) and 20 Asian (*Elephas maximus*); this represented 46% of the total UK captive elephant population of 63 animals. The elephants ranged from 2–44 years of age and the mean age was 22 years. Twelve were born in the wild, the remaining 17 were born in captivity.

### Statement of ethics

The study involved observational assessment of captive elephant behaviour, with no disruption to their behaviour or routine. The project was approved by the Ethics Committee at the University of Nottingham, School of Veterinary Medicine & Science, and by the ethics committees of each of the five participating safari parks and zoos.

### Design of the prototype welfare assessment tool

#### Identification of welfare indicators

**Fig 1:**
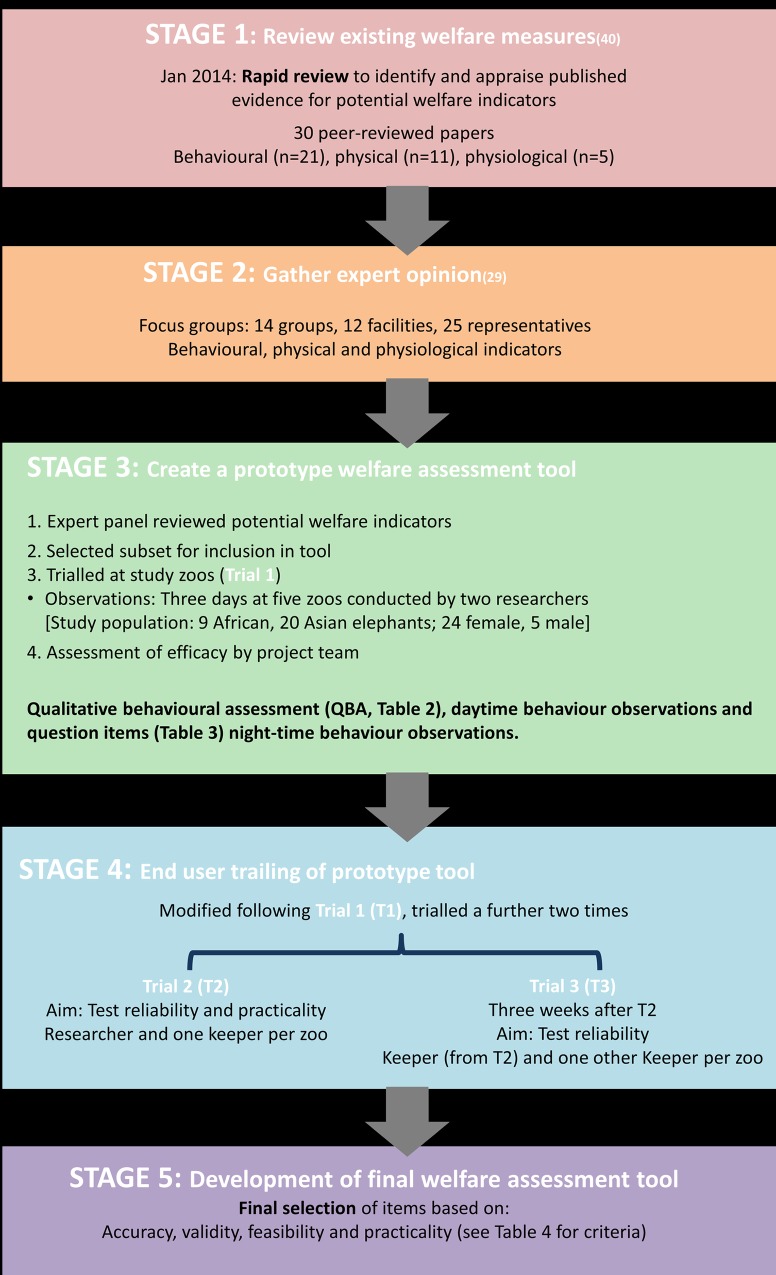
Overview of development process of welfare assessment tool.

Elephant behavioural welfare indicators were identified from: 1) a rapid review of peer reviewed literature using a systematic search criteria and a critical appraisal tool (see [19]); 2) a review of non-peer reviewed publications on elephant welfare (see [41]); and 3) stakeholder focus groups to identify novel measures (see [30]). Inclusion of keepers in the focus groups meant that some of the indicators included in the tool were suggested by end users; such inclusive participation when developing welfare assessments is considered best practice [17]. Seventy-four potential indicators of welfare were identified by the focus groups, forty one were identified from the peer reviewed literature and a further seventy eight from non-peer reviewed literature. These measures were combined into a summary list for consideration by an external advisory panel, consisting of people working in zoo management, and researchers in animal welfare and in behaviour of captive or free living elephants. Duplicate measures were removed as were those which were not considered behavioural measures of welfare. A list of 76 unique behavioural measures of welfare was produced for potential incorporation in the tool. See Asher and colleagues (2015) for full details of the categories. A full outline of tool development can be found in Appendix A in S1 Appendix, and a brief summary in Fig 1.

#### Welfare assessment tool

The list of 76 welfare indicators was then considered for inclusion in the prototype tool. Final selection of measures for inclusion was based on the strength of evidence of their validity or importance as welfare measures (see [19]), their feasibility and practicality for use by elephant keepers and the need to provide a range of measures across different aspects of welfare. The welfare assessment tool was designed to take no longer than 60 minutes to complete.

The prototype welfare assessment tool consisted of three parts (see Appendix B in S1 Appendix for full prototype tool):

1 **Qualitative Behaviour Assessment:** Determining the valence of an animal’s emotional or affective state has been identified as an important aspect of welfare assessment [42–44]. Qualitative behavioural assessment (QBA) is a methodology which was developed to capture this dimension of animal welfare through assessment of an animal’s demeanour [26–29]. Sixteen terms (depressed, active, fearful, indifferent, engaged, distressed, exploratory, social, content, relaxed, uncomfortable, agitated, tense, frustrated, wary, and playful) were scored by participants on a visual analogue scale (VAS) (Table 2), completed four times in the day; each scoring was based on demeanour observed during a one-minute live observation. One 1-minute live observation had to take place in each of four 2-hour time blocks: (1) 9:00–11:00 am; (2) 11:00 am– 1:00 pm; (3) 1:00 pm– 3:00 pm; and (4) 3:00 pm– 5:00 pm) so that observations were spread throughout one full day. A mean score for each term was generated from the ratings of that term at each of the four time points.

**Table 2:** QBA terms used in welfare tool and anchors for VAS. Proposed welfare interpretation, based on valence of term is indicated by a + (positive) or–(negative).

Term	Definition	Anchors
Content +	Appears at ease, tranquil, seems satisfied.	Not content/ content
Depressed -	Seems lethargic, uninterested in physical environment or social companions, unwilling to engage when solicited, head posture hunched or slumped.	Not depressed/ depressed
Relaxed +	Peaceful, seems free from tension.	Not relaxed/ relaxed
Uncomfortable -	Ill at ease without a clear context for any distress. Body, trunk, head postures un-relaxed and possibly changing frequently, appear fidgety.	Comfortable/ uncomfortable
Fearful -	Poised as if ready to flee, anticipatory defensive postures with ears, head and body. Head and trunk up, possibly in defensive herd star shape.	Not fearful/fearful
Agitated -	A state of uncertainty which can be accompanied by physical restlessness and over-reaction to stimuli e.g. trumpeting. Scanning environment in a tense and anxious fashion.	Not agitated/ agitated
Tense -	Body, head, trunk, held in a rigid fashion, un-relaxed reactions to stimuli.	Not tense/tense
Frustrated -	Reacting to seeking a goal without success; can be violent (kicking, tusking, whacking with trunk, head pushing with body, head-on charge) towards others or objects or take the form of tossing objects about as a displacement activity. Angry body posture.	Not frustrated/frustrated
Wary -	Sometimes nervous, paused reaction to some stimuli, unwilling to move in or out of an area, may be accompanied by listening and smelling. It is a slow and calm behaviour.	Not wary/wary
Playful +	Engaged in a bout of object, locomotor or social play. Responds positively to solicitations for play.	Not playful/playful
Attentive +	Appears interested in the environment and/or engaged with objects or individuals, has a generally positive demeanour.	Indifferent/ attentive
Distressed (upset) -	Animals seems to be suffering from a loss, may search the environment restlessly or without apparent purpose. May be accompanied by head shakes frequent distress rumbles or bellows	Not distressed/distressed

2 **Daytime behaviour Questions**: Keepers were asked to score 35 questions on Likert or VAS (Table 3) following three days of live observations. Five-minute long observations were undertaken four times per day (one 5-minute observation in each of four 2-hour time blocks spread across the day as described above), and were repeated over three consecutive days. The daytime behaviour questions were scored at the end of the third day of live observations. Likert scales for behavioural frequency were used where appropriate, with different numbers of response options based on the expected frequency of that behaviour (based on pilot data and initial keeper feedback).

**Table 3:** Prototype Daytime behaviour questions, question text, answer options and indication of proposed relationship to welfare with supporting references.

Section	Question text	Answer options	Option(s) proposed to indicate worse welfare
1.Stereotypies	This elephant has performed a stereotypy …	6 point Likert scale ranging from ‘never’ to ‘always’ and higher scores indicate worse welfare	Higher scores (more stereotypy) [19, 30]
	If you have seen this elephant stereotype please give a breakdown of the stereotypies seen, their approximate frequency and the approximate time of day they occurred	Descriptive	Descriptive information for zoo records
	Do the stereotypies this elephant performs ever interfere with their behaviour?	5 point Likert scale ranging from ‘stereotypies do not interrupt flow of behaviour’ to ‘stereotypies frequently disrupt intended action’	Higher scores (more disruption of intended action which may also indicate association with historical rather than current events) [30, 45, 46]
	Can the stereotypies this elephant performs be interrupted?	Multiple choice and descriptive	Less easy to disrupt (may also indicate association with historical rather than current events) [30, 45]
2.Comfort behaviour	This elephant has dust bathed…	7 point Likert scale ranging from ‘almost every time I looked at them’ to ‘never’	Lower scores (less dustbathing) [19]
	This elephant has rolled in sand…	7 point Likert scale ranging from ‘almost every time I looked at them’ to ‘never’	Lower scores (less rolling) [19]
	This elephant has wallowed…	3 point Likert scale ranging from ‘more than once a day during my observations’ to ‘never’	Lower scores (less wallowing) [19]
	This elephant has interacted with water features (pools, fountains, showers or similar)… In a few words please describe the interaction and type of water feature when most of this interaction occurred	7 point Likert scale ranging from ‘almost every time I looked at them’ to ‘never‘ and descriptive	Lower scores (less interaction with water) [19, 30]
3.Feeding	I have seen this elephant feeding …	6 point Likert scale ranging from ‘less than daily’ to ‘almost every time I looked at them’	A sudden change: higher feeding [19] and lower feeding [19, 30]
	I have seen this elephant …	VAS with anchors ‘Rarely forage and/or only feed at scheduled feed times’ to ‘Forage for food all the time it is free to do so’	Lower scores (less spontaneous foraging) [19, 30]
4.Walking	This elephant was walking (but not pacing) during its free time…	6 point Likert scale ranging from ‘almost every time I looked at them’ to ‘less than daily’	Lower scores (less walking) [19]
5.Activity	I have seen this elephant …	VAS with anchors ‘Spend most of its day waiting for scheduled events’ to ‘Engaging in activities completely independent of the scheduled events’	Lower scores (more time waiting for scheduled event and less spontaneity in behaviour) [29]
	This elephant was standing still (but not resting)…	7 point Likert scale ranging from ‘almost every time I looked at them’ to ‘never‘	Higher scores (more standing still) [19]
6.Social and environmental interactions	I have seen this elephant interacting with the environment (investigating or interacting with things in the environment other than food with the trunk … In a few words please describe with what they were interacting	7 point Likert scale ranging from ‘almost every time I looked at them’ to ‘never‘ and descriptive	Lower scores (less interaction with environment) [19, 30]
	I have seen this elephant …	VAS with anchors ‘avoid other elephants every time it is free to do so’ to ‘spend time near or approach other elephants every time it is free to do so’	Lower scores (more avoidance of conspecifics) [19, 30]
	I have seen this elephant engaging in affiliative behaviour (any positive social interaction, e.g. touching another elephant in a non-aggressive manner) … In a few words please describe with whom they were interacting and how	7 point Likert scale ranging from ‘never‘ to ‘almost every time I looked at them’ and descriptive	Lower scores (less affiliative behaviour) [19, 30]
	I have seen this elephant engaging in agonistic behaviour (any negative social interaction, behaving in a manner which causes harm or potential harm to conspecifics, e.g. displaces, displays, chases, bites) … In a few words please describe with whom they were interacting and how	7 point Likert scale ranging from ‘almost every time I looked at them’ to ‘never‘ and descriptive	Higher scores (more agonistic behaviour) [19, 30]
	I have seen this elephant engaging in object play (throwing or kicking debris or an object around in a playful interaction. This can include environmental enrichment) … In a few words please describe the interaction with the object	3 point Likert scale ranging from ‘never’ to ‘more than once a day’ and descriptive	Lower scores (less object play) [19, 30]
	I have seen this elephant playing with conspecifics (engaging in active play with another elephant, including head to head sparring, trunk wrestling, mounting, chasing, and rolling on one another. Does not include behaviours observed following an antagonistic encounter or as part of courtship) … In a few words please describe with whom they were interacting and how	3 point Likert scale ranging from ‘never’ to ‘more than once a day’ and descriptive	Lower scores (less playing) [19, 30]
7.Important observations	Please provide details of other observations you believe are of importance	Descriptive	For information only
8.General experience from working with the elephant	When was the last time you saw this elephant come across a new or unexpected situation? What was the situation and what was their reaction?	Descriptive	For information only
	Vocalisations and contexts	Descriptive	For information only
9. Overall welfare	At this current point in time please assess the mental health, physical health and overall welfare of this elephant	VAS ranging from ‘worst imaginable’ to ‘best imaginable for any elephant anywhere’	Lower scores

3 **Night-time observations:** Keepers were asked to score night time behaviour from video footage using scan sampling every 30 -minutes for one night, from 21:00–09:00 (or whatever time keepers arrived in the morning), during the three day observation period. Behaviour was scored as: Feeding, standing or lying (alone or with others), stereotypy, walking, comfort, interaction with environment, social, other (with a space to write in what behaviour, not already listed, was observed), or out of view. Of these measures: feeding, walking, comfort, interaction with environment, social, and standing or lying with others were proposed to indicate positive welfare; standing or lying alone or performing stereotypy were proposed to indicate negative welfare (based on[29, 40])

### Welfare assessment tool trial and subsequent development

To test the practicality and feasibility of use the tool was trialled at three time points by a researcher and keeper: Trial 1) by two researchers [including EW]; Trial 2) by a single researcher [EW] and at least one keeper from that zoo (November–December 2014) to test inter-observer reliability (by both people observing the same elephants at the same time); Trial 3) by the same keeper from Trial 2 at each zoo (at least three weeks after Trial 2) to test for intra-observer reliability, as well as one additional keeper at each zoo (to assess inter-observer reliability). After the first trial, the tool was modified; measures which could not be easily rated accurately were removed, and additional options were added in for the answers where required. The tool was then used in Trials 2 & 3 to gather further input from elephant keepers, and to assess the reliability and validity of final measures. All keepers were briefed on use of the tool prior to undertaking these trials.

#### Recording equipment

Bespoke video cameras with infrared capability were used to make recordings of both the indoor and outdoor enclosures, except when facilities had existing indoor cameras (in which case these were used for indoor footage). The cameras were high definition Hikvision IR network cameras (Model DS-2CD2632D-IS, Hikvision Europe, The Netherlands), customised to run from battery power (Tracksys, Nottingham, UK) and were mounted on pre-existing structures at each facility when possible, or on bespoke 3 meters steel stands (Oryx Engineering and Installation, UK), at locations which provided fullest visual coverage of the enclosures. Cameras recorded at 20FPS and had a 20m IR light range. Two additional 40 metre, 80 degree angle IR lamps (Camsecure, Bristol, UK) were mounted on the stand for each camera at 90° relative to each other (and 45° to each side of the camera), to provide wider IR coverage at night.

#### Reliability and validity testing

Analysis was performed to assess the validity, reliability and feasibility of the prototype monitoring tool, and to identify groupings of elements of the tool in order to reduce the number of measures being analysed. In order to analyse the accuracy of representation of the welfare assessment, during Trials 1 & 2, video footage of the elephants was collected over three consecutive 24 hour periods and was scored using a detailed ethogram (see Appendix C in S1 Appendix). Generally, all behaviours were assessed in both daytime and night time footage. However, there were a few exceptions. Swimming and bathing was only possible during the daytime, so this was only included in the daytime ethogram. Proximity to others (within 3 body lengths of another elephant) was only included in the daytime ethogram, as the size of the night time enclosures may have led to a false interpretation of elephants being proximate to one another when they were just in the same enclosure. Running was also included in the daytime but not the night time ethogram, as smaller night time space often precluded this behaviour. Daytime footage (09:00–17:00) was analysed using five minute scan sampling and night-time footage (18:00–08:00) was analysed using three minute scan sampling for all behaviour except standing and lying rest, which were recorded continuously. Sampling frequency was tested to ensure that timing of scan samplings provided an accurate reflection of behaviour (when it was sampled more frequently) for both daytime and night-time ethogram observations. Five and three minute sampling, respectively, were compared to one minute sampling and found to sufficiently capture frequency of behaviour. The more frequent sampling at night reflected the sampling rate required for capturing social behaviour which was additionally recorded, but is not presented here. While detailed behaviours were captured in the ethograms, analysis of behaviours was made using the higher level behavioural categories of the behaviours from the ethogram, in order to compare the results to the welfare assessment tool.

Face validity and feasibility of the tool were assessed using keeper and expert feedback. Inter-rater reliability was assessed by comparing scores of the researcher and the keeper on Trial 2 at each zoo, for each element of the tool. Test re-test reliability was assessed by comparing scores by the keepers at each zoo involved in Trial 2 assessment with scores by that same keeper at each zoo for Trial 3. Internal consistency and groupings of questions were identified. Concurrent criterion validity was tested to confirm that behavioural indicator of welfare as assessed by the tool did indeed measure the behaviour it was intended to measure. This was achieved by comparing the keeper responses to questions about reported frequency of behaviour in the daytime using the tool with detailed ethogram analysis of video recordings from Trial 2. For night-time observations the frequencies at which behavioural indicators of welfare were observed from night-time observations made by keepers were compared to the proportion of observations of those same behaviours in detailed ethogram analysis of video recordings from Trial 2. Cut-off criteria and analysis performed for each type of reliability or validity were assigned prior to analysis (see Table 4).

**Table 4:** Overview of data analysis and criteria for assessing reliability and validity of the welfare tool.

Test	QBA	Questions	Night observations
**Face validity (and feasibility)**	Keeper and expert feedback		
**Data reduction and internal reliability/consistency**	Exploratory Principle Components Analysis (PCA) to determine potential groupings which were tested using Cronbach’s alpha (criteria > 0.6)		Not completed
**Test re-test/Intra-rater reliability**	For continuous scores: Bland Altman statistics (criteria <6% points outside limits of agreement); For ordinal data: Kappa coefficients (>0.4)		
**Concurrent criterion validity**	Not done	For each behavioural indicator which passed criteria of reliability, the scores collected in the tool were compared to those from an in depth ethogram analysis. General Linear Models (GLMS) were applied with the proportion of time in relevant behaviour from ethogram analysis as outcome variable and element of tool as predictor. For night time observations, data was compared per night and ‘night’ (matched or not) was included as a fixed effect and interaction. Rare behaviour was converted to a binary scale. Criteria: element of tool is significant (P<0.05) predictor of relevant behaviour measured by ethogram.	
**Known groups criterion validity**	Compared groups of elephants previously suggested to have better or worse health, welfare or longevity. Scores on the questionnaire were considered in terms of quartiles, except QBA scores which were kept as raw scores and scores which had a binomial distribution were collapsed to a binary score. GLMS or binary logistic regressions were performed as appropriate. Univariate analysis followed by forward stepwise selection was applied. The following were seven predictors were considered: Body Condition Score (henceforth BCS), Foot health score, Gait score, any health problems experienced in the previous 12 months, the number of inter-zoo transfers they had experienced, whether they were related to any other group members, the elephant’s origin (i.e. captive-born or wild-caught). Not used to makes decisions about which items to include in the tool.		

We collated data from BIAZA’s Elephant Welfare Group on: body condition score (henceforth BCS, noting higher, rather than lower BCS are more generally a welfare concern due to problems with captive elephant obesity); foot health score; gait score; any chronic or acute health conditions experienced in the previous 12 months; whether they were related to any other group members; the number of inter-zoo transfers they had experienced; the elephant’s origin (i.e. captive-born or wild). These variables were used for known-groups criterion validity because welfare manipulation was not possible in this context. Each of these variables is related to health (BCS, foot health, gait score and health conditions) or has been associated with welfare in other studies: the number of inter-zoo transfers [7, 45], relatedness [23], and the elephant’s origin [7, 47].

All analysis was conducted in the statistical programme R [48] using packages stats: BlandAltmanLeh, psy, psych, polcor, lme4.0, and lmerTest.

### Final welfare assessment tool

#### Identification of indicators

Final selection of indicators for inclusion in the welfare assessment tool was based on the strength of evidence of their validity as welfare indicators, their feasibility and practicality for use by elephant keepers, their accuracy as compared to thorough behavioural analysis and the desire to include a range of measures across different areas of welfare to create a more robust tool. A few questions were included for future interest but not analysed. These were questions on vocalisations (because stakeholders and expert panel believed they were reflective of welfare but there was little current evidence to support this in elephants) and an overall welfare assessment score (scored on a VAS from Worst imaginable and to Best imaginable for any elephant anywhere). The purpose of the overall welfare assessment score was to provide information on individual welfare for zoo records which may not have been captured by the other aspects of the tool.

## Results

### Qualitative behaviour assessment

Some QBA terms could be combined into component groupings, but terms ‘Playful’ and ‘Wary’ did not group easily with other terms. One component which emerged was labelled: ***At ease in the environment*** which was comprised of higher ratings on ‘Content’ and ‘Relaxed’, and lower ratings of ‘Uncomfortable’, ‘Agitated’, ‘Tense’ and ‘Frustrated’. Cronbach’s alpha revealed good internal reliability for this grouping component (0.90). This component was found to be reliably completed on different occasions and by different raters, as were two additional QBA terms, ‘Playful’ and ‘Wary’ (see Table 5). The QBA terms were not validated against detailed behavioural recordings, but they were analysed for known-group validity. Using this analysis elephants were found to be rated as more wary if they had experienced a health problem in the previous 12 months (by 0.77±0.38, t = 2.03, P = 0.05).

**Table 5:** Reliability statistics for the three parts of the behavioural welfare tool.

	Statistics for inter-rater reliability RA 2 vs keepers 2		Statistics for test retest keepers 2 vs keepers 3		
Element of welfare tool (range / maximum)	% of points outside limits of agreement	mean /critical difference *	% points outside limits of agreement	mean /critical difference*	Reliability accepted †
**QBA**					
Distressed (0–6.5/10)	4%	1.06/2.13	9%	0.43/3.14	NO
Fearful (0–9.4/10)	2%	1.10/2.46	7%	0.42/3.40	NO
Attentive(0-10/10)	6%	1.08/4.17	4%	0.33/7.80	NO
Playful (0-10/10)	5%	1.13/3.13	5%	0.41/3.08	YES
Depressed (0–6.6/10)	7%	1.01/2.62	6%	0.39/2.95	NO
Wary (0–6.6/10)	6%	1.02/2.79	5%	0.13/2.91	YES
*‘At ease with the environment’*	2%	1.12/2.69	4%	0.22/3.43	YES
**Day-time questions**					
1.1. Stereotypy (1-4/6)		*0.56		*0.80	YES
2.1. Dustbathing (1-5/7)		*0.85		*0.03	NO
2.2. Sand rolling (1-5/7)		*0.85		*0.74	YES
2.4. Water interaction (1-4/7)		*0.35		*0.40	NO
4.1 Walking (1-5/6)		*0.04		*0.38	NO
5.2. Standing still (1-6/7)		*0.09		*0.40	NO
6.2. Avoid others (1.6-10/10)	3%	0.44/2.40	3%	0.63/3.13	YES
6.4. Agonistic(1-5/7)		*0.58		*0.62	YES
6.5. Object play(1-3/3)		*0.41		*0.55	YES
Dependence on routine(0.20.7/1)	0%	<0.01/0.17	0%	0.03./0.21	YES
Engaging with environment (0.1–0.8/1)	0%	0.19/0.24	0%	0.10/0.27	YES
Activity (0.1-1/1)	8%	0.18/0.49	3%	0.05/0.34	NO
**Night-time observations**			** **	** **	** **
Feeding observations (0–0.9/1)	0%	<0.01/0.21	6%	0.04/0.61	YES
Standing rest others (0–0.7/1)	0%	0.02/0.25	6%	0.07/0.46	YES
Standing rest alone (0–0.5/1)	0%	0.02/0.12	25%	0.07/0.46	NO
Lying rest near others (0–0.7/1)	0%	0.02/0.31	0%	0.02/0.31	YES
Lying rest alone (0–0.5/1)	0%	0.04/0.20	0%	<0.01/0.12	YES
Walking (0–0.3/1)	7%	0.01/0.12	15%	0.15/0.13	NO
Stereotypy (0–0.3/1)	0%	<0.01/0.08	0%	0.02/0.05	YES
Social behaviour (0–0.3/1)	0%	<0.02/0.18	7%	0.04/0.16	NO
Interaction Environment (0–0.7/1)	3%	0.02/0.11	3%	0.04/0.16	YES
Longest lying rest (0–330)			0%	18.46/123.66	YES

*Kappa used for ordinal variables

^†^ Reliability accepted if for both inter-rater and test retest <6% points outside limits of agreement for continuous data OR Kappa coefficients >0.4 for ordinal data.

### Daytime behaviour questions

Three groupings of daytime behaviour questions were identified. The first grouping, labelled ***Dependence on routine***, comprised questions on: Feeding frequency (higher), Feeding at scheduled time only (higher), Waiting for scheduled events (higher) and Playing with others. (lower) The internal reliability of this grouping of questions as assessed by Cronbach’s alpha was 0.67. Based on proposed interpretation of the individual items, higher dependence on routine was proposed as a negative welfare indicator. A second grouping labelled ***Positively engaging with the physical and social environment*** consisted of: Wallowing frequency, Interactions with the environment, and Affiliative behaviour (all higher). This grouping’s internal reliability, as assessed by Cronbach’s alpha, was 0.68. Based on proposed interpretation of the individual items, higher positive engagement was proposed as a positive welfare indicator. A final grouping related to ***Activity*** consisted of: Walking frequency (higher) and Standing still frequency (lower) and the internal reliability, as assessed by Cronbach’s alpha, was 0.82. Based on proposed interpretation of the individual items, higher activity was proposed as a positive welfare indicator.

Out of twelve questions assessed for test-retest/intra- and inter-rater reliability, four questions did not reach an acceptable level of reliability (see Table 5). These were Interaction with water, Walking frequency, Dustbathing and Standing still frequency.

The majority of questions were found to be associated with the relevant behaviour observed in the ethogram analysis of behaviour, providing concurrent criterion validity for these questions to confirm they are measuring the behaviour they were designed to measure (see Table 6). Exceptions to this were Sand rolling and Object play.

**Table 6:** Concurrent validity statistics for the daytime and night-time behavioural observations part of the behavioural welfare tool.

Element of tool	Validated against ethogram analysis	Statistics from GLMs	Validity accepted
**Day-time questions**			
1.1. Stereotypy	Proportion Stereotypy	t = 2.75, P = 0.012	YES
2.2. Sand rolling	Proportion Maintenance behaviour	t = 2.01, P = 0.057	NO
2.3. Wallowing	Proportion Wallowing	t = 5.14, P<0.001	YES
3.1. Feeding	Proportion feeding	t = 2.72, P = 0.012	YES
3.2. Feed outside schedule	Proportion feeding	t = 2.29, P = 0.032	YES
6.1. Interact(ion) environment	Proportion Interaction environment	t = 2.04, P = 0.045	YES
6.2. Avoid others	Proportion of time within proximity of 3 elephant lengths or less to others	t = 3.60, P = 0.033	YES
6.3. Affiliative	Proportion of time engaged in presumed affiliative behaviour	t = 2.42, P = 0.024	YES
6.4. Agonistic	Proportion of time engaged in presumed agonistic behaviour	t = 2.39, P = 0.026	YES
6.5. Object play	Proportion of time engaged in object play	t = 1.37, P = 0.185	NO
6.6. Play others	Proportion of time engaged in play with others	t = 2.98, P = 0.007	YES
**Night-time observations**			
Feeding observations	Proportion of observations feeding*	t = 3.31 P = 0.002	YES
Standing rest others	Proportion of observations standing rest others	t = 2.26 P = 0.040	YES
Lying rest near others	Proportion of observations lying rest others	t = 3.05 P = 0.008	YES
Lying rest total	Proportion of observations lying rest	t = 2.85 P = 0.012	YES
Stereotypy	Presence or absence of stereotypy*	t = 5.32 P<0.001	YES
Agonistic behaviour	Proportion of observations agonistic behaviour	t = 2.79 P = 0.014	YES
Longest period lying rest	Longest bout of lying rest*	t = 2.29 P = 0.041	YES
Environment interaction	Presence or absence of environmental interactions	t = 2.12, P = 0.034	YES

* significance based on matched night

***Dependence on routine*** (which included answers to questions on Feeding frequency, Feeding at scheduled times, Waiting for scheduled events and less playing with others) was positively associated with (worse) foot health scores (0.45 ±0.09, t = 4.84, P<0.001) and gait scores (0.016±0.05, t = 3.27, P = 0.003). The question on stereotypy frequency, which had a binomial distribution, was associated with gait score and whether elephants were related to other members of the herd. Elephants were more likely to show more stereotypy if: (i) they had higher (worse) gait scores (OR = 1.66, CI = 1.01–2.74, P = 0.047); (ii) they were not housed with related herd members (OR = 22.97 CI = 1.53–34.37, P = 0.02).

### Night-time observations

Ten elements of the night-time observations section of the welfare tool could be assessed for reliability; of these, seven met the criteria for being reliable (see Table 5). Some behaviour types recorded in the night-time observations (Comfort behaviour and Interactions with the environment) were so rare that they could not be assessed for reliability. Standing rest alone, Walking and Social behaviour, which were recorded as part of the prototype welfare tool, were not reliable between or within raters. Feeding, Standing rest with others, Lying rest (alone or with others), Stereotypy and Length of the longest lying bout, could be assessed reliably.

The relative proportion of eight out of eight behaviour types at night assessed using the welfare tool were representative of the relative proportions of these same behaviours using detailed ethogram video analysis (see Table 6).

The length of the longest lying bout was more likely to be shorter if elephants (i) did not have any health problems (more likely to score in 1^st^ Quartile, 5.67, CI = 1.73–18.6, P = <0.001); (ii) had a lower BCS (more likely to score in 1st Quartile, OR = 1.61, CI = 1.02–2.56-, P = 0.05); or (iii) had a higher foot score (more likely to score in 2^nd^ Quartile OR = 1.35, CI = 1.82–3.30, P = 0.01 and in 1^st^ Quartile, OR = 4.75, CI = 2.31–9.76, P<0.001).

### Finalised tool

Based on the results from the prototype tests, a finalised Elephant Behavioural Welfare Assessment Tool was developed for use by captive elephant managers (see Appendix D in S1 Appendix). All those elements of the tool which met the criteria for reliability and validity were included (see Tables 5 and 6 and Table 7). The finalised tool is presented to keepers in an Excel sheet in which they enter the raw data and see the final scores. Those questions which can be grouped together to form a single score are automatically rotated as necessary and averaged to form a single score for each component (Positively engaging with the physical and social environment; Dependence on routine; At ease in the environment) which is then presented to the keepers as an outcome of the tool alongside the single scores for all other non-groupable questions.

**Table 7:** Content of final elephant behavioural welfare tool.

Section	Type of Observation	Time Period Covered	Question Types	Information Gathered /Content of Questions
*Section A* Qualitative Behavioural Assessment—Daytime	Live	One day Four 1-minute observations, spread across day	Rating using Descriptive Adjectives	Content, Relaxed, Uncomfortable, Agitated, Tense, Frustrated, Wary, Playful,
*Section B* Daytime Activity	Live	Three days On each of 3 days, conduct four 5-minute observations, spread across the day (make daily notes, complete questionnaire end of third day)	Multiple choice and Visual Analogue Scale (VAS)	Stereotypies, Wallowing, Feeding, Activity, Social and Environmental Interactions, Response to Unexpected Situations, Vocalisations, Welfare Ratings (mental and physical health, and overall welfare)
*Section C* Night-time Activity	Review video footage	One night Record behaviours observed for each elephant every 30 minutes throughout night period. Plus continuous sampling for any instances aggression in night.	Checkmark on data sheet if behaviour seen by that elephant at each time period. Identify elephants with whom lie down; identify elephants with whom show agonistic behaviour	Stereotypies, Lying down (and with whom or if alone), Feeding/foraging, Interacting with the Environment, Comfort (self-maintenance), Social behaviour. Any instances of aggression (time, behaviour, elephants involved)

## Discussion

This project involved the development of a novel, evidence-based, behavioural welfare assessment tool for use in evaluating the welfare of captive elephants. The behavioural welfare assessment tool developed in this project was designed to address a specific need in the elephant-holding zoo community in the UK: the need for a validated (as far as possible in the time available and constraints of research in a zoo environment), relatively rapid, easy-to-use tool that could be regularly used by elephant keepers to make behavioural assessments of welfare. The results of the study suggest that this aim was successfully met, as many behavioural indicators of welfare, previously validated as such from other studies, could be reliably scored using the tool designed. Furthermore, many of the indicators measured using the tool were representative of that behaviour measured using a gold standard ethological method of scoring behaviour every 3–5 minutes for 72 hours. A number of behavioural indicators of welfare assessed during the day using the tool were closely matched to the ethological behaviour scoring; these included: feeding, wallowing, stereotypy and play behaviour. At night, agonistic behaviour and lying rest, particularly when the lying rest occurred near others, were both measured accurately using the tool, as these measures also closely matched the results from the ethological behaviour scoring. An excel spreadsheet with pre-designed formulae, and drop down boxes, has been designed and distributed to the zoos for ease of data entry and collation and interpretation of results. This will allow zoos to assess the impact of changes in management and husbandry, to facilitate evidence-based management of their elephants, and is available from the authors on request.

The aim of this study was not dissimilar to those of the AssureWel and AWIN projects [49, 50], both of which involved development of practical on-farm welfare assessments of farmed species. Like the current study, the AWIN project also used behaviour as a central part of their welfare assessments and used stakeholder input to develop more user-friendly protocols [50]. Similar to the current project, AWIN also incorporated QBA in their welfare assessment for some species. Unlike the current study, both AWIN and AssureWel included assessments of health and physical condition in addition to behaviour. There are other projects and protocols developed by BIAZA’s Elephant Welfare Group (EWG) which have been designed to assess these aspects of welfare [51]. Used together it is hoped that these tools will provide a more complete overview of UK zoo elephant welfare.

A number of approaches have been suggested for assessing animal welfare, and it was possible to incorporate some, but not all, of these considerations in the newly developed tool described here. This new tool will provide some preliminary indications of elephant preferences: in where, how and with whom they choose to spend their time, within the constraints of their environment. This may help elephant-holding facilities to identify with which, of the resources elephants have available, they most interact. Facilities can share best practise, however, this will always be limited to what is available to elephants across different facilities, and time spent interacting with resources does not always indicate preference for that resource.

QBA was used in the tool and may have potential to capture the valence of the elephants’ emotional state. Although we were not able to validate this, we have demonstrated that some QBA terms can be rated reliably by keepers and some terms are associated with physical welfare state. Further validation is needed against other measures or manipulations of emotional state. Indeed, complete validation of a welfare tool is an extensive process and cannot be completed in a single study. Future work should explore potential links between behavioural scores made in this tool in better and worse environments and in comparison against specific positive or negative welfare outcomes (e.g. health parameters, or reproductive activity).

One other aspect of welfare which is sometimes considered to be important is telos, which has been defined as ‘nurturing and fulfilment of the animal’s nature’ [52]. Assessment of telos, or of natural behaviour, in captive elephants might best be accomplished through comparisons with wild elephant behaviour (time budgets, physical activities, social groups) [23], although others argue against this approach, favouring instead a focus on the consequences of behaviour [53]. This newly developed tool assesses a wide range of natural elephant behaviours seen in both captive and wild elephants (including comfort behaviour, sleep, foraging, social interactions, exploratory behaviour).

### Proposed welfare tool elements: Elements requiring further consideration

Overall, the sample size was sufficient to allow assessment of validity and reliability against pre-determined criteria (without correction for multiple testing). There were a small number of males in the the dataset (20%), however, this is largely reflective of the UK elephant population where only ~25% are adult males. Whilst our sample was highly representative, covering 49% of the UK elephant population, the numbers are still relatively small from a statistical testing perspective. As a result it was not felt appropriate to correct for multiple testing, and as different comparisons explore different aspects of validity/reliability, the number of tests conducted within a single comparison were reduced to the minimum needed. Although the number of comparisons made here were extensive, this is in line with guidelines for the development of new psychometric tools [54], which require testing for internal consistency, criterion validity, and reliability. If correction for multiple testing had been used, wallowing measured during the daytime and stereotypy and lying near others at night-time as measured by the tool would have remained significantly associated with ethological measurements of this same behaviour. Evidence of replication of the results with a new dataset will be tested in the coming years as the tool is used over time.

In general, the majority of the elements tested in the prototype tool met the criteria for reliability and validity, and were therefore included in the final version of the tool (see Appendix D in S1 Appendix). There were, however, some measures which were not reliable or not valid in all contexts, and further work is needed to either investigate other ways to assess those particular aspects of behaviour, or to determine the meaning or significance of variations in the expression of these behaviours. There was poor agreement between reports of water interaction and object play recorded using the tool, and ethological assessments of these behaviours. It is possible that this is because these behaviours occurred rarely, so the less frequent behavioural assessments made when using the tool were less likely (by chance) to occur at the right time to detect the performance of such behaviour. Movement or activity levels (walking or standing still) were not accurately assessed in the prototype tool and questions on these were therefore removed from the final tool. Alternative methods for assessing this behaviour should be sought in the future, as activity can be an important part of physical welfare [55, 56]. The proposed QBA term ‘depressed’ was not used reliably by different assessors and feedback by keepers indicated that they found it a difficult term to use. Alternative terms such as ‘lethargic’ or ‘apathetic’ have been suggested to capture this dimension. It is not entirely clear how to best collect data on night time behaviour; a number of measures were reliable and reflected behaviour measured on that particular night, but did not necessarily reflect behaviour over a longer time frame (such as the three nights across which the tool was validated). It may be worth extending the night time observations over more nights, but more would need to be known about the consistency of such behaviour at night before this could be recommended.

Results from this study suggested the interpretation of one of the behavioural indicators of welfare used in the tool may need to be reconsidered. Prior to undertaking the study, it had been assumed that longer bouts of lying rest were indicative of better welfare, as an absence of lying rest is associated with poor welfare [57]. However, it is possible that longer, uninterrupted bouts of lying rest might also be an indicator of poor welfare since in the current study, this was associated with health problems, and with poorer foot and gait scores (and associated with higher BCSs, suggesting that higher body weight may have contributed to these issues). In general, the known-groups analysis conducted was not used to select measures for inclusion in the tool, however this analysis provided some interesting initial results. Only four of the items in the tool (including one composite measure of four questions) were associated with the known-groups which were health related measures or circumstances which had previously been related to welfare (relatedness of herd, zoo transfers, origin). The fact that more items were not associated with these variables is not unexpected due to small sample sizes, large individual variance between elephants, and the known groups being related only to certain aspects of welfare (hence why known-groups was not a criteria for inclusion in the tool). However, it must be noted that many of the items still require criterion validation from more suitable measures (e.g. ideally a welfare manipulation within an individual). The items for which known-groups validation was supported generally fitted expectations with regard to direction of relationships with welfare. The QBA component ‘wary’ was higher in elephants that had experienced a health problem in the past 12 months; although it is important to note that keepers would have had knowledge of this and this may have influenced their QBA rating. Stereotypic behaviour was higher in animals which were not housed with related herd mates. More stereotypy was also associated with worse gait which could either be explained by stereotypy influencing gait, or some shared experience which influenced both gait and stereotypy. Higher ‘Dependence on routine’ was associated with poorer foot and gait health. This mirrors findings in dairy cows, which show higher levels of routine (i.e. visiting the same feeder and stall every day) when they are lame [58].

This tool was not intended to compare welfare of different elephants at different facilities; it would not be appropriate to do so for a number of reasons. A number of the measures in this tool, including most notably stereotypies, represent an animal’s cumulative welfare state, rather than their current welfare. Furthermore, there are a range of individual factors, such as life history, health status, age, and reproductive status among many others, which can influence the results from this welfare tool, and it is important to take these into account when interpreting results. This tool has instead been designed to monitor changes in an individual elephant’s behavioural welfare over time, and to assess the impact of changes in management and husbandry. Changes in husbandry and management might include, for example, access to a new enclosure or environmental enrichment, a new type of flooring, a new elephant added to the collection, or a move of an elephant to a new facility.

## Conclusions

This study describes the development of a new elephant behavioural welfare assessment tool designed to be relatively rapid, reliable and easy to use, to facilitate regular use by elephant keepers. To date the tool has been used by 11 UK and Irish facilities, and many of these facilities have used it multiple times to begin to track possible changes in welfare over time. The tool comprises three sections: (1) Qualitative behaviour assessment, rating demeanour of the elephant on 12 terms, scored after four sets of 1-minute observations across one day; (2) A series of questions answered after four sets of 5-minute daytime behaviour observations across three days; (3) Night-time observations, consisting of reviewing overnight footage, and recording behaviour using 30 minute scan sampling for one night.

Regular use of this tool by captive elephant facilities is recommended (e.g. quarterly) to facilitate assessment and monitoring of elephant welfare over time. This information can be used to determine the impact of any changes in husbandry and management of elephant welfare, and can help facilities to develop and adjust individual elephant welfare plans to optimise the welfare for each elephant in their care.

We would suggest that the methodology used for this project could similarly be employed to develop and validate behavioural welfare assessment tools to evaluate welfare in a wide range of species in a zoo or aquarium setting. This would enable a more comprehensive approach to monitoring the welfare of these species over time, and determine response to changes in management and husbandry, to better understand the impact of management decisions and to better inform policy to support optimal zoo animal welfare.

## Supporting information

S1 Appendix (DOCX)

S1 Data (XLSX)
