# Supplementary material for: Development of a behavioural welfare assessment tool for routine use with captive elephants
Source: PLoS One. 2019 Feb 6;14(2):e0210783. doi: 10.1371/journal.pone.0210783 (PMC6364905; doi:10.1371/journal.pone.0210783)
Supplement: S1 Appendix — (DOCX) [file pone.0210783.s001.docx]

Supporting information

## Appendix A in S1 Appendix: Stages in development of a Behavioural Welfare Assessment Tool for Routine Use with Captive Elephants

1. **Identify potential behavioural indicators of welfare.** Elephant-specific behavioural measures of welfare were identified from three sources: 27 indicators were identified from peer reviewed literature (Williams et al. in press), 78 from grey literature (Asher et al. 2015) and 55 from teleconferences held with stakeholders (Chadwick et al. 2017). After considering synonyms a total of 76 measured remained. Consideration of other practical welfare assessments in other species led to inclusion of QBA. 16 QBA terms were included based on consultation with Françoise Wemelsfelder who designed QBA for welfare assessment and Phillis Leigh, who together had previously designed terms and trialled QBA in wild elephants.
2. **Review of behavioural measures of welfare by project Expert Advisory panel**. The panel considered 89 behavioural measures written on separate pieces of paper. They grouped similar measures according to what they were measuring as: Social interactions, Abnormal Repetitive Behaviours (ARBs), Arousal/anticipatory Behaviours, Comfort (self-maintenance), Activity budgets, Environmental interactions, Cognitive measures, Vocalisations, Qualitative. The panel combined measures which they considered to be part of the same behaviour and measures believed to be functionally equivalent. They excluded measures if they could not be consistently applied across all elephants: those which related to human-elephant interactions which differ across zoos and contact styles; those which related to infants being present or related to mating. They excluded measures with no clear link to welfare and Qualitative measures which it was believed would be captured by QBA. Cognitive measures were excluded because there was no possibility to measure these by pure observation.

The 89 measures were: Behavioural Synchrony with group, Group cohesion, Consistency of Associations, Trunk position (exploratory or sensory), Nursing, Affiliative behaviours, Proximity (close approach – within one elephant length), Play (with conspecifics), Touching temporal gland, Trunk interaction (trunk to trunk or trunk to body), Allomothering, Food sharing, Agonistic behaviours, Displacement (approach/avoid/supplant from resource), Avoidance, Aggression, Pacing/route tracing, Trunk swinging, Tusk banging, Head banging, Rocking, Bar biting, Swaying/weaving, Self-directed ARBs (e.g. pulling teat), Pawing, Head bobbing, Trunk sucking, Alert, Head position, Ear position and movement, Foot swinging, Temporal gland secretion, Pedalling/trampling, Movement of tail, Inactive, Dust bathing, Washing, Use of pool, Mud wallowing, Swimming, Ear flapping, Scratching, Masturbation, Self-directed interaction, Change from ‘normal’ behaviour, Active/inactive, Locomotion, Standing, Feeding, Eating, Foraging, Drinking, Resting, Lying rest, Enrichment use, Object manipulation, Exploration, Responsiveness, Interaction with substrate, Manipulation of the environment, Digging, Object play, Preference/aversion, Tool use, Speed of learning, Response to novelty, Behaviours occurring under stress, Excessive aggression, Monitoring environment (olfactory, auditory, visual), Clustering/bunching, Startle/vigilance response, Rumble, Loud vocalisations, Trumpet, Greeting, Let’s grumble, Play vocalisations, Sexual vocalisations, Aggressive vocalisations, Contentment, Apathy/depression, Relaxed, Attitude, Alert, Looking ‘happy’, Looking ‘guilty’, Posture (alert, wary or relaxed), Facial expression.

1. **Final selection of indicators to measure.** Measures were assessed for inclusion in the prototype welfare assessment tool based on the strength of evidence of their validity or importance as welfare measures, their feasibility and practicality for use by elephant keepers and to ensure inclusion of a range of measures across different areas of welfare.

The indicators the tool was designed to measure were: Affiliative behaviour (to cover measures of Touching temporal gland, Trunk interaction, trunk to trunk or trunk to body), Proximity to conspecifics, Play (with conspecifics), Agonistic behaviours, Aggression, Anticipation, Stereotypy (with type specified to cover Pacing/route tracing, Trunk swinging, Tusk banging, Head banging, Rocking, Bar biting, Swaying/weaving, Self-directed ARBs, Pawing, Head bobbing, Trunk sucking), Inactive, Comfort/Self maintenance behaviour (to include Dust bathing, rolling in sand, wallowing, interaction with water), Use of water and wallowing, Locomotion, Feeding and Foraging, Resting specified as Lying or Standing rest and near conspecifics or not, Environmental interaction to capture (Enrichment use, Object manipulation, Exploration, Interaction with substrate, Manipulation of the environment, Digging). Aspects of demeanor/qualitative expression of behaviour using QBA. Vocalisations were also included but only for future exploratory purposes as there was high agreement from panel and stakeholder consultation that these were indicative of welfare but have been little researched.

1. **Design first pre prototype tool**. 51 parts of the pre prototype welfare assessment tool were designed: 16 QBA terms; 24 questions on daytime behaviour; 11 measures of night-time behaviour. A range of measures were incorporated using question types previously demonstrated to be effective for use in other practical assessments of welfare and to attempt to minimise the time needed to complete the tool. The tool was designed to take no longer than 30 minutes to complete per elephant assessed.

**Trial 1. Pre prototype tool included:**

**QBA**: Observation of animals for five minutes - QBA questions then answered according to this time 16 terms [depressed, active, fearful, indifferent, engaged, distressed, exploratory, social, content*, relaxed*, uncomfortable*, agitated*, tense*, frustrated*, wary*, playful* ]

*included in final welfare assessment tool.

**Day time behaviour questions**: Observation of elephants for 3 days followed by:

Create the following three pie charts to provide an approximate percentage time spent in the following activities: Pie 1: stereotypies, feeding/foraging, walking, comfort (self maintenance), inactive, other; Pie 2: social behaviour: alone, negative interaction, positive interaction, neutral interaction; Pie 3: breakdown of different types of stereotypical behaviour for this animal

Answer questions on:

Time of day of stereotypies

Vocalisations (including contexts)

Rate frequency of Interaction with environment (VAS - never to ‘almost always’) & Affiliative behaviour (VAS - always alone to almost always with at least one other member of the herd)

Describe situation when: Least social interaction occurs; Most social interaction occurs

Object play (never engages in object play to almost always…) & Describe situation when:

Least object play occurs; Most object play occurs

Play with conspecifics (never to almost always…) & Describe situation when: Least conspecific play occurs; Most conspecific play occurs.

Aggressive behaviour

Night-time behaviour observations: based on video footage obtained from zoos. Behaviour scored included: Feeding, Sleeping or resting while standing and alone, Sleeping or resting while lying and alone, Sleeping or resting while standing and with others, Sleeping or resting while lying and with others, Stereotypy, Walking, Comfort, Interaction with Environment, Social, Other (describe), Out of view and Aggression.

1. Trial 1: completed by research assistant [EW] and one of two research assistants [RA]. Data gathered by RA was used to assess initial face validity and reliability between EW and RA. Tool was trialled at all study zoos to test feasibility of assessment. Video camera recordings were made at the same time as observations. Observations were carried out over three days. The pre-prototype was shown to keepers for discussion.

Feedback from trial 1 QBA: Five minutes was too long to create a ‘snapshot’ e.g. if an altercation occurred that may affect demeanour for a proportion of the five minutes but wouldn't necessarily represent the full five minutes. This was subsequently reduced to 1 minute 4 times per day. Terms which caused ambiguity in interpretation or were difficult to assess were identified. E.g. ‘social: soliciting interaction with other or responding positively to social interactions from others’ was removed because there was no option to record an elephant as just having no other elephants near it without recording it as ‘anti-social’. Reaction to conspecifics may also have changed depending upon which conspecific was engaged in the interaction. This data was also captured in the latter sections of the welfare assessment tool. Questions: Terminology was modified to capture subtle interactions which may have been missed - e.g. aggressive was changed to antagonistic - because overt aggression was very rare whilst small agonistic interactions may have been more frequent and it was important that they weren’t missed. Pie charts were identified as too difficult to produce. This was initially modified to just calculating rough percentages. Following trial 1 it was decided to remove this question and reformulate as multiple choice questions.

1. **Modify the pre-prototype based on trial 1 to develop a testable prototype.** Upon completion of trial 1 modifications were made to the tool. The project team together considered evidence and experiences from trail 1 to remove measures which could not be reliably rated in the time frame, or which were not representative of behaviour being undertaken. Likert scales were edited according to how frequently behaviour occurred. An overall welfare score was added to allow for any aspect of welfare not covered by the tool’s questions to be incorporated into the tool.
2. **Prototype trialled by end users and researcher in Trial 2**. EW and a keeper (one keeper per zoo), Completed the prototype tool as outlined in Appendix C. Further feedback from keepers sought. Video recordings made of elephants to assess the validity of the tool in measuring the requisite behaviour.
3. **Second trial of prototype Trial 3 by more than one keeper per zoo.** This allowed the tool to be conducted without the presence of EW to ensure it could be completed without further support and to test inter-rater/ test- retest reliability: Due to the time between trials (at least 3 weeks) both would be tested by comparing results from Trial 2 & 3.
4. **Create final tool.** Modification of tool based on results of reliability, validity and keeper feedback to create final welfare assessment tool. Detailed in the main manuscript.
5. **Improve use of ease and record keeping of tool.** Excel spreadsheet developed to ease use of tool and records kept.
6. **Continued Improvement and validation of tool.** Use of tool by 11 UK and Irish facilities. Multiple uses by some facilities to begin to process of tracking possible changes in welfare over time. Use of tool quarterly recommended but also requested during times of change or potential welfare compromise (e.g. relocation to new zoo) to build evidence relevant to validity.

## Appendix B in S1 Appendix: Prototype Elephant Behavioural Welfare Assessment Tool

This document was given to keepers at each of the five trial zoos. It consisted of instructions, and the paperwork to be completed.

**Keeper Instructions:**

The welfare assessment tool is broken down into three broad areas:

1. Qualitative Behavioural Assessment
2. Daytime activity
3. Night-time activity

### Qualitative Behavioural Assessment

Designed to gather subjective data about the current welfare state of each individual. This section is designed to be used as a point of comparison in future assessments, allowing for documentation of change over time. Observations should be conducted throughout the day on the first day of data collection (this will be on Tuesday - see data collection timetable below). Following each observation we would ask keepers to complete a short assessment of the individual. Observations must not be made within 30 minutes of an event which may substantially affect behaviour (e.g. feeding or being let inside/outside). If more than two keepers are assessing each elephant then both keepers must be conducting observations at the same time.

### Daytime activity

Designed to gather objective data on the day-time activity for each individual. Five minute observations (for a maximum of 4 elephants observed at a time) should be conducted at least four times per day (see data collection timetable below). On Thursday afternoon we would ask keepers to answer a series of questions about the activity of the individual(s) observed. Specific questions will be asked about the occurrence of the following activities:

- Stereotypies
- Comfort behaviours (e.g. dust bathing, rolling in sand, wallowing, interactions with water sources)
- Feeding
- Walking
- General activity
- Social and environmental interactions (e.g. interaction with the environment, time spent with conspecifics, affiliative and agonistic behaviour and play with objects and conspecifics)

### Night-time Activity

Designed to gather objective data of the night-time activity for each individual. The research technician and keepers will observe over-night video footage of the indoor enclosures.

Scan sampling (recording behaviours observed at set times throughout a sampling period, e.g. every 30 minutes) will be used to document the frequency of the following behaviours:

- Stereotypies
- Sleep
- Feeding
- Interacting with the environment
- Walking
- Comfort (self-maintenance)

Focal sampling (recording all occurrences of a behaviour throughout a sampling period) will be used to document the frequency of the following behaviour:

- Excessive aggression

## Prototype Tool

| **Section 1 – Qualitative Behavioural Assessment** | |
| --- | --- |
| **Keeper Tool** |  |
| Observe animals for one minute 4 times throughout the day (2 in the morning and 2 in the afternoon) – answer a series of QBA questions based on this short observation  **Notes:**   - 1 minute observations must be made 4 times per day (2 in the morning and 2 in the afternoon). One observation must be made in each of the following time blocks:  1. 09:00 – 11:00 2. 11:00 – 13:00 3. 13:00 – 15:00 4. 15:00 – 17:00  - Observations must not be made within 30 minutes (either before or after) of an event which may affect behaviour, e.g. feeding or being let inside/outside | Keepers to make a note by each term whether they like or dislike and why. There is a box in the left margin by each term. Please put a tick or cross in each box to indicate whether you like/dislike the term. |

**Live Observations – Qualitative Behavioural Assessment**

| Elephant: | Date: Time: | Weather: |
| --- | --- | --- |

Please complete this short Qualitative Behavioural assessment based on **1 minute** of live observation of this individual.

***Content:*** *appears at ease, tranquil, seems satisfied*

| Not content |  | Content |
| --- | --- | --- |

***Depressed:*** *seems lethargic, uninterested in physical environment or social companions, unwilling to engage when solicited, head posture hunched or slumped.*

| Not depressed |  | Depressed |
| --- | --- | --- |

***Relaxed:*** *Peaceful, seems free from tension.*

| Not relaxed |  | Relaxed |
| --- | --- | --- |

***Uncomfortable:*** *ill at ease without a clear context for any distress. Body, trunk, head postures un-relaxed and possibly changing frequently, appear fidgety.*

| Comfortable |  | Uncomfortable |
| --- | --- | --- |

***Fearful:*** *Poised as if ready to flee, anticipatory defensive postures with ears, head and body. Head and trunk up, possibly in defensive herd star shape.*

| Not fearful |  | Fearful |
| --- | --- | --- |

***Agitated:*** *a state of uncertainty which can be accompanied by physical restlessness and over-reaction to stimuli e.g. trumpeting. Scanning environment in a tense and anxious fashion.*

| Not agitated |  | Agitated |
| --- | --- | --- |

***Tense:*** *body, head, trunk, held in a rigid fashion, un-relaxed reactions to stimuli.*

| Not tense |  | Tense |
| --- | --- | --- |

***Frustrated:*** *reacting to seeking a goal without success; can be violent (kicking, tusking, whacking with trunk, head pushing with body, head-on charge) towards others or objects or take the form of tossing objects about as a displacement activity. Angry body posture.*

| Not frustrated |  | Frustrated |
| --- | --- | --- |

***Wary:*** *sometimes nervous, paused reaction to some stimuli, unwilling to move in or out of an area, may be accompanied by listening and smelling. It is a slow and calm behaviour.*

| Not wary |  | Wary |
| --- | --- | --- |

***Playful:*** *engaged in a bout of object, locomotory or social play. Responds positively to solicitations for play.*

| Not playful |  | Playful |
| --- | --- | --- |

***Attentive:*** *appears interested in the environment and/or engaged with, objects or individuals, has a generally positive demeanour.*

| Indifferent |  | Attentive |
| --- | --- | --- |

***Distressed (upset):*** *Animals seems to be suffering from a loss, may search the environment restlessly or without apparent purpose. May be accompanied by head shakes frequent distress rumbles or bellows*

| Not distressed |  | Distressed |
| --- | --- | --- |

| **Section 2 – Daytime activity** | |
| --- | --- |
| **Keeper Tool** |  |
| Based on the last 3 consecutive days with these elephants (Tuesday to Thursday of data collection week) please answer the questions below.  **Notes:**   - 5 minute observations* must be made at least 4 times per day (distributed throughout the day). At least one observation must be made in each of the following time blocks:  1. 09:00 – 11:00 2. 11:00 – 13:00 3. 13:00 – 15:00 4. 15:00 – 17:00   *5 minute observations per 2 elephants observed   - During the observation period the focal elephant(s) must be followed around to ensure a clear view is maintained wherever possible | Keepers to make a note by each term whether they like or dislike each question and why. There is a box in the left margin by each term. Please put a tick or cross in each box to indicate whether you like/dislike the question. |

**Live Observations – Daytime activity**

**Please answer the following questions based on the live observations you have made over the last 3 days (Tuesday to Thursday of data collection week):**

| Elephant: | Date: Time: | Weather: |
| --- | --- | --- |

**Section 1: Stereotypies**

- 1. During the last 3 days of observations this elephant has performed a stereotypy...

1. Always (few, if any, stereotypy-free intervals)
2. Very frequently (virtually every hour)
3. Frequently (daily, multiple times per day)
4. Occasionally (daily but infrequent)
5. Rarely (not daily)
6. Never
   1. If you have seen this elephant stereotype please give a breakdown of the stereotypies seen, their approximate frequency and the approximate time of day they occurred

| Description | Frequency (using above scale) | Time of day | Location |
| --- | --- | --- | --- |
|  |  |  |  |
|  |  |  |  |
|  |  |  |  |
|  |  |  |  |
|  |  |  |  |

- 1. Do the stereotypies this elephant performs ever interfere with their behaviour?

1. Stereotypies do not interrupt flow of behaviour
2. Stereotypies occasionally interrupt flow of behaviour
3. Stereotypies frequently interrupt flow of behaviour
4. Frequently interrupt flow of behaviour and occasionally disrupt intended action
5. Stereotypies frequently disrupt intended action
   1. Can the stereotypies this elephant performs be interrupted? Yes/No/Unknown

If yes, please describe what they are interrupted by:

**Section 2: Comfort Behaviour**

- 1. During the last 3 days of observations this elephant has dust bathed…

1. Almost every time I looked at them
2. Most but not every time I have looked at them
3. Half or more of the times I have looked at them
4. Less than half the times I have looked at them
5. Around once a day during my observations
6. Less than daily during my observations
7. Never during my observations
   1. During the last 3 days of observations this elephant has rolled in sand…
8. Almost every time I looked at them
9. Most but not every time I have looked at them
10. Half or more of the times I have looked at them
11. Less than half the times I have looked at them
12. Around once a day during my observations
13. Less than daily during my observations
14. Never during my observations
    1. During the last 3 days of observations this elephant has wallowed…
15. More than once a day during my observations
16. Around once a day during my observations
17. Never during my observations
    1. During the last 3 days of observations this elephant has interacted with water features (pools, fountains, showers or similar)…
18. Almost every time I looked at them
19. Most but not every time I have looked at them
20. Half or more of the times I have looked at them
21. Less than half the times I have looked at them
22. Around once a day during my observations
23. Less than daily during my observations
24. Never during my observations

In a few words please describe the interaction and type of water feature when most of this interaction occurred

**Section 3: Feeding**

- 1. During the last 3 days of observations I have seen this elephant feeding...

1. Almost every time I looked at them
2. Most but not every time I have looked at them
3. Half or more of the times I have looked at them
4. Less than half the times I have looked at them
5. Around once a day during my observations
6. Less than daily during my observations
   1. During the last 3 days of observations I have seen this elephant...

| Rarely forage and/or only feed at scheduled feed times |  | Forage for food all the time it is free to do so |
| --- | --- | --- |

**Section 4: Walking**

- 1. During the last 3 days of observations this elephant was walking (but not pacing) during its free time…

1. Almost every time I looked at them
2. Most but not every time I have looked at them
3. Half or more of the times I have looked at them
4. Less than half the times I have looked at them
5. Around once a day during my observations
6. Less than daily during my observations

**Section 5: Activity**

- 1. During the last 3 days of observations I have seen this elephant...

| Spend most of its day waiting for scheduled events |  | Engaging in activities completely independent of the scheduled events |
| --- | --- | --- |

- 1. During the last 3 days of observations this elephant was standing still (but not resting)…

1. Almost every time I looked at them
2. Most but not every time I have looked at them
3. Half or more of the times I have looked at them
4. Less than half the times I have looked at them
5. Around once a day during my observations
6. Less than daily during my observations
7. Never during my observations

**Section 6: Social and Environmental Interactions**

- 1. During the last 3 days of observations I have seen this elephant **interacting** **with the environment** (investigating or interacting with things in the environment **other than food** with the trunk...

1. Almost every time I looked at them
2. Most but not every time I have looked at them
3. Half or more of the times I have looked at them
4. Less than half the times I have looked at them
5. Around once a day during my observations
6. Less than daily during my observations
7. Never during my observations

In a few words please describe with what they were interacting

- 1. During the last 3 days of observations I have seen this elephant...

| Avoid other elephants every time it is free to do so |  | Spend time near or approach other elephants every time it is free to do so |
| --- | --- | --- |

- 1. During the last 3 days of observations I have seen this elephant engaging in **affiliative behaviour** (any positive social interaction, e.g. touching another elephant in a non-aggressive manner) ...

1. Almost every time I looked at them
2. Most but not every time I have looked at them
3. Half or more of the times I have looked at them
4. Less than half the times I have looked at them
5. Around once a day during my observations
6. Less than daily during my observations
7. Never during my observations

In a few words please describe with whom they were interacting and how

- 1. During the last 3 days of observations I have seen this elephant engaging in **agonistic behaviour** (any negative social interaction, behaving in a manner which causes harm or potential harm to conspecifics, e.g. displaces, displays, chases, bites) ...

1. Almost every time I looked at them
2. Most but not every time I have looked at them
3. Half or more of the times I have looked at them
4. Less than half the times I have looked at them
5. Around once a day during my observations
6. Less than daily during my observations
7. Never during my observations

In a few words please describe with whom they were interacting and how

- 1. During the last 3 days of observations I have seen this elephant **engaging in object play** (throwing or kicking debris or an object around in a playful interaction. This can include environmental enrichment) ...

1. More than once a day during my observations
2. Around once a day during my observations
3. Never during my observations

In a few words please describe the interaction with the object

- 1. During the last 3 days of observations I have seen this elephant **playing with conspecifics** (engaging in active play with another elephant, including head to head sparring, trunk wrestling, mounting, chasing, and rolling on one another. Does not include behaviours observed following an antagonistic encounter or as part of courtship) ...

1. More than once a day during my observations
2. Around once a day during my observations
3. Never during my observations

In a few words please describe with whom they were interacting and how

**Section 7**

Please provide details of any other observations you have made of this animal in the last 3 days which you believe are of importance

**Section 8**

**Please answer the following questions based on your general experience from working with this elephant:**

- 1. When was the last time you saw this elephant come across a new or unexpected situation? What was the situation and what was their reaction?
  2. Please describe in the table below any vocalisations this individual has made and the contexts in which they occurred

| Vocalisation – Rumble, Trumpet or Other (please provide short description) | Context |
| --- | --- |
|  |  |
|  |  |
|  |  |

**Section 9**

**At the current point in time...**

Please place a mark, line or circle along the line where you feel is appropriate for each aspect of health and welfare for this individual.

- 1. **Mental health**

Worst imaginable

Best imaginable for any elephant anywhere

- 1. **Physical health**

Worst imaginable

Best imaginable for any elephant anywhere

- 1. **Overall Welfare**

Worst imaginable

Best imaginable for any elephant anywhere

**Comments**

**Definition of Terms for Behaviour Ethogram**

**Sleeping/Resting: A rapidly reversible state of immobility and greatly reduced sensory responsiveness, an inactive state where no obvious activity is being performed**

- **Standing rest**: Upright and stationary with 3 or 4 feet on the ground. Not performing any other behaviour. Eyes may be closed. End of trunk usually curled on floor. Individual may be leaning on an object (e.g. enclosure bars, a wall or a sand pile) or conspecific.
- **Lying rest**: Lateral recumbence, no other behaviours are being performed

**Stereotyping: Repetitive behaviour with no obvious purpose. May include but is not limited to the following:**

- **Head bobbing**: backward and forward or in an arc, walking in circles
- **Swaying/weaving**: Side to side or back and forth repetitive swaying of the body
- **Pacing**: Walking repeatedly along the same route in an unvarying, repetitive pattern
- **Leg swing**: Standing still repeatedly swinging one front leg back and forth
- **Foot lifting**: Standing still repeatedly lifting one foot in the air
- **Rocking**: Rocking back and forth transferring weight from hind to front legs
- **Tusk banging**: Repetitive banging or rubbing of the tusks on objects (e.g. enclosure bars or logs)
- **Bar biting**: Chewing or gnawing on enclosure bars

**Comfort: Any self-maintenance or grooming behaviour. May include but is not limited to:**

- **Rubbing**: Rubbing the body against an object
- **Wallowing**: Lying down and rolling in mud
- **Rolling**: Lying down and rolling in dirt or sand
- **Scratching**: Scratching the body with trunk or foot - this can include using the trunk to scratch/feel gently around the skin, eye or ear
- **Scratching with tool**: Scratching the body with a tool, e.g. branch or stick
- **Throwing straw on self**: Throwing soft objects such as grass or straw onto the body using the trunk
- **Dust bath**: Spraying mud, dust or sand on the body
- **Water bath**: Spraying water on the body
- **Body slap**: Hitting of own body with tail or trunk, appears to be a means of removing an insect or other irritant

**Walking: Taking two or more steps in any direction in a non-repetitive pattern. Only one foot is removed from the ground at any one time.**

**Social: Any positive or neutral interaction with another elephant. May include but is not limited to:**

- **Leaning**: Leaning on another elephant
- **Standing**: Standing underneath or standing above another elephant
- **Trunk-mouth**: Putting the trunk in the mouth of another elephant
- **Touching**: Touching another elephant with the trunk in a non-aggressive manner
- **Tail**: Holding the tail of another elephant with the trunk or underneath a leg
- **Trunk-trunk**: Intertwining of trunks between two elephants
- **Approach**: Moving in a non-aggressive manner, within one body length of another elephant
- **Climb**: Placing at least one foot on top of another elephant - usually one that is lying down
- **Offer food**: One elephant pushes a pile of food towards another elephant, looks like an offering of the resource
- **Trunk lifting**: Trunk is outstretched and raised towards an approaching individual
- **Sitting on an elephant**: Sitting in a crouched position on top of another elephant which is in lying rest
- **Rubbing elephant**: Rubbing the body against another elephant
- **Playing with an elephant:** Engaging in active play with another elephant, including head to head sparring, trunk wrestling, mounting, chasing, and rolling on one another. Does not include behaviours observed following an antagonistic encounter or as part of courtship

**Interaction with the environment: investigating or interacting with things in the environment (other than food). May include but is not limited to:**

- **Playing with an object**: Throwing or kicking debris or an object around in a playful interaction
- **Object manipulation**: Examination or manipulation of an object with the trunk and/or foot
- **Environmental investigation**: Investigating things in the environment (other than food) with the trunk - looks like the individual may be 'sniffing' at the ground or objects

**Feeding: the process of locating and consuming food stuffs**

- **Eating**: Manipulation (including breaking up of food stuffs using the trunk or foot) and consumption of food
- **Foraging**: The process of searching for and collecting food stuffs using the trunk and/or foot - may include kicking up grasses, shaking the food in the trunk or beating against the leg

**Checklist of Overnight Behaviours Observed**

Name of elephant: Date:

| **Time** | **Behaviours Observed** | | | | | | | | | |
| --- | --- | --- | --- | --- | --- | --- | --- | --- | --- | --- |
|  | **Feeding** | **Sleeping or resting** | | **Stereotypy** | **Walking** | **Comfort** | **Interaction with Environment** | **Social** | **Other (describe)** | **Out of view** |
|  |  | **Standing or Lying** | **Alone or with others** |  |  |  |  |  |  |  |
| **21:00** |  |  |  |  |  |  |  |  |  |  |
| **21:30** |  |  |  |  |  |  |  |  |  |  |
| **22:00** |  |  |  |  |  |  |  |  |  |  |
| **22:30** |  |  |  |  |  |  |  |  |  |  |
| **23:00** |  |  |  |  |  |  |  |  |  |  |
| **23:30** |  |  |  |  |  |  |  |  |  |  |
| **00:00** |  |  |  |  |  |  |  |  |  |  |
| **00:30** |  |  |  |  |  |  |  |  |  |  |
| **01:00** |  |  |  |  |  |  |  |  |  |  |
| **01:30** |  |  |  |  |  |  |  |  |  |  |
| **02:00** |  |  |  |  |  |  |  |  |  |  |
| **02:30** |  |  |  |  |  |  |  |  |  |  |
| **03:00** |  |  |  |  |  |  |  |  |  |  |
| **03:30** |  |  |  |  |  |  |  |  |  |  |
| **04:00** |  |  |  |  |  |  |  |  |  |  |
| **04:30** |  |  |  |  |  |  |  |  |  |  |
| **05:00** |  |  |  |  |  |  |  |  |  |  |
| **05:30** |  |  |  |  |  |  |  |  |  |  |
| **06:00** |  |  |  |  |  |  |  |  |  |  |
| **06:30** |  |  |  |  |  |  |  |  |  |  |
| **07:00** |  |  |  |  |  |  |  |  |  |  |
| **07:30** |  |  |  |  |  |  |  |  |  |  |
| **08:00** |  |  |  |  |  |  |  |  |  |  |
| **08:30** |  |  |  |  |  |  |  |  |  |  |
| **09:00** |  |  |  |  |  |  |  |  |  |  |

**Comments**

## Appendix C in S1 Appendix. Daytime and Night time Ethograms for Captive Elephants

| **Category** | **Behaviour** | **Definition** | **Only Included in Daytime (Day) Ethogram** |
| --- | --- | --- | --- |
| **Stereotypical** | Rocking | Repetitive bobbing of the head and/or body |  |
|  | Weaving | Side to side or back and forth repetitive swaying of the body |  |
|  | Trunk tossing | Vigorous swaying of trunk from side to side |  |
|  | Head pressing | Pressing head up against an object with no obvious purpose |  |
|  | Pacing | Walking repeatedly along the same route in an unvarying, repetitive pattern |  |
|  | Leg swing | Standing still repeatedly swinging one front leg back and forth |  |
|  | Foot lift | Standing still repeatedly lifting one foot in the air |  |
|  | Weight transfer | Rocking back and forth transferring weight from hind to front legs |  |
|  | Tusk banging | Repetitive banging or rubbing of the tusks on objects (e.g. enclosure bars or logs) |  |
|  | Bar biting | Chewing or gnawing on enclosure bars |  |
|  | Throwing faeces | Tossing faecal material into air or on self |  |
|  | Linear pacing | Taking a few steps forward then tracing the route by taking a few steps backward. Believed to be initially caused by being chained over prolonged periods by a front and back leg simultaneously; an elephant will walk forwards until restrained by the back leg chain and then walk backwards until restrained by the front leg chain. |  |
| **Anticipatory** | Loitering | Standing alert, often near (within two elephant body lengths) of gates or enclosure bars prior to an event (e.g. feeding, moving inside, etc.) |  |
| **Maintenance & Comfort** | Wallowing | Lying down and rolling in mud, dirt or sand |  |
|  | Throwing straw on self | Throwing soft objects such as grass or straw onto the body using the trunk |  |
|  | Dust bath | Spraying mud, dust or sand on the body |  |
|  | other | shake, rub, stretch, scratch, scratch with tool, body slap |  |
|  | Trunk in own mouth | Holding trunk in own mouth - a behaviour frequently seen in young elephants |  |
|  | Trunk under body | Holding trunk under own body |  |
|  | Own tail | Holding own tail in trunk |  |
| **Interaction with Water** | Bathe | Standing or lying in water up to the depth of the belly or deeper | Day |
|  | Spray body | Spraying water on the body |  |
|  | Spray out | Spraying water outwards using the trunk |  |
|  | Clearing water | Moving the trunk backwards and forwards across a water surface prior to drinking |  |
|  | Swimming | Movement within a body of water where the feet are not touching the bottom | Day |
|  | Playing with a water source | Engaging in active play with a water source, may involve rolling around within a body of water or manipulating a water source with the trunk |  |
|  | Other |  |  |
| **Feeding/ Foraging/Drinking** | Eating or Wadge | Manipulation (including breaking up of food stuffs using the trunk or foot) and consumption of food; or wadge (Chewing of food stuffs to extract juice and sugars, not accompanied by ingestion) |  |
|  | Foraging | The process of searching for and collecting food stuffs using the trunk and/or foot - may include kicking up grasses, shaking the food in the trunk or beating against the leg |  |
|  | Drinking | Taking water straight into the mouth, usually kneeling at the edge of a water body - more common in calves |  |
|  |  | Water is drawn into the trunk and then sprayed into the mouth - more common in juveniles and adults |  |
| **Locomotion** | Walk | Taking two or more steps in any direction in a non-repetitive pattern. Only one foot is removed from the ground at any one time. The way an elephant 'walks' may be affected by many factors, e.g. reproductive status or the context of the situation, i.e. during conflict (see relevant sections for more details) |  |
|  | Exaggerated walk | As with 'walk' but head and trunk movements become more pronounced, the head moves up and down with the rhythm of the walk and the trunk will swing side to side |  |
|  | Run | A faster paced version of walking; more than one foot is removed from the ground at any one time. | Day |
|  | Other (including spin, side step) | Spin: Occurring on land or in water; the rotation of the body in a circular motion. |  |
|  |  | Side step: A sideways movement, achieved by the elephant crossing one leg over the front of the other |  |
|  | Standing | Upright and stationary, 3 or 4 feet on the ground. |  |
| **Resting** | Standing rest | Upright and stationary with 3 or 4 feet on the ground. Not performing any other behaviour. Eyes may be closed. End of trunk usually curled on floor. Individual may be leaning on an object (e.g. enclosure bars, a wall or a sand pile) or conspecific. |  |
|  | Lying rest | Lateral recumbence, no other behaviours are being performed |  |
|  | Clearing | Clearing an area (usually prior to lying rest), wiping sand with the trunk, moving objects or faeces out of the way |  |
|  | Changing sides | Changing from lying rest on one side to the other without fully standing |  |
| **Interaction with Environment** | Object throw | Using the trunk to throw an object at another elephant or person |  |
|  | Object manipulation | Examination or manipulation of an object with the trunk and/or foot - often seen when an elephant is manipulating environmental enrichment to gain access to a food reward |  |
|  | Environmental investigation | Investigating things in the environment (other than food) with the trunk - looks like the individual may be 'sniffing' at the ground or objects |  |
|  | Tool usage | Using an object to help the elephant obtain an immediate goal, e.g. using logs to gain access to food resources |  |
|  | Tusking | Digging with tusks into ground, or rubbing of tusks on logs |  |
|  | Digging | Kicking or scraping dust or dirt backward/behind the elephant. May also occur as an aggressive behaviour, or prior to engaging in lying rest |  |
| **Social** | Affiliative touch (with trunk) | trunk in mouth of other,touching any area other, holding tail of other, intertwining trunks, |  |
|  | Affiliative touch (not with trunk) | leaning on another, rubbing body against other,nudge other elephant, sit or stand on other |  |
|  | Trunk lifting | trunk outstretched and raised towards an approaching individual |  |
|  | Back toward | Elephant turns to present posterior and walk slowly backwards into another individual |  |
|  | Parallel walk | Two elephants walking side by side in a non-aggressive manner, for 3 or more steps |  |
|  | Follow | One elephant walks closely behind (within 2 elephant body lengths) of another elephant |  |
|  | Offer food | One elephant pushes a pile of food towards another elephant, looks like an offering of the resource |  |
|  | Approach | Moving in a non-aggressive manner, within one body length of another elephant |  |
|  | Standing | Standing over another elephant - usually one that is lying down or young |  |
| **Agonistic** | Tusking | Poking or jabbing at another elephant with the tusk |  |
|  | Charge | Move towards another elephant with the head held high, pace usually quickens as individual gets closer to the target elephant, can lead to pursuit of another elephant |  |
|  | Bite | Biting of the body, trunk or tail of another elephant |  |
|  | Kick | Strike out or hit an elephant or object with a foot in a seemingly aggressive manner - note object may include enclosure bars or kicking of sand towards another elephant |  |
|  | Strike | Hitting another elephant with the trunk or tail |  |
|  | Push | One elephant forces or pushes against the body (usually the rump) of another elephant, resulting in the elephant that is being pushed moving at least two steps |  |
|  | Stand off | Two elephants standing facing in opposite directions with foreheads pushing against each other |  |
|  | Smack | Hitting the trunk on the floor in an aggressive manner, may be accompanied by a 'snort' |  |
|  | Food stealing | Taking food from another elephant |  |
|  | Directed trunk swing | head oriented towards another elephant, human or change in the environment, violently swinging the trunk around in an aggressive display |  |
|  | Aggressive display: standing | Facing another elephant in an aggressive posture; head held high, ears wide or flapping |  |
|  | Aggressive display: walking | Display of dominance while walking; head bobbing up and down or side to side, ears wide or flapping |  |
|  | Redirected aggression | During a conflict situation an individual may redirect their aggression onto another individual or object, e.g. uprooting trees or throwing objects |  |
|  | Size up | Two elephants directly facing each other, standing as tall as possible, heads raised and ears spread wide |  |
|  | Intervening | An elephant places its body between the aggressor and the target (i.e. 'protecting' the target elephant from the attack) |  |
|  | Directed ear fold | Head oriented towards another elephant, human or change in the environment, ears extended, horizontal fold or crease across the middle of the ear |  |
|  | Directed ear flap | Head oriented towards another elephant, human or change in the environment, ears held out perpendicular to the head, flapping |  |
|  | Freeze | Head oriented towards another elephant, human or change in the environment, no movements are occurring, elephants looks 'alert' |  |
|  | Lunge | A lunging motion followed by physical contact, used to prevent another elephant standing up |  |
|  | Avoidance | Avoiding or shying away from elephants or people; the individual turns their head, rump or whole body away from the particular elephant or person |  |
|  | Retreat | Avoiding or shying away from elephants or people; the individual either walks forwards away from or backwards away from a particular elephant or person |  |
|  | Displacement | Movement of one elephant results in another elephant leaving its location (within 10 seconds) - usually occurs when a more dominant elephant approaches a more subordinate individual |  |
|  | Chase | Charge leading to pursuit of another elephant |  |
|  | Resource holding | Elephant with a food source turns its rump to an approaching elephant to create a block between the approaching elephant and the food source |  |
|  | Pushing food | Pushing food around with trunk or foot - usually done when an elephant is moving a food resource away from an approaching elephant |  |
| **Play** | Playing with an object | Throwing or kicking debris or an object around in a playful interaction. Sometimes more than one elephant will engage in play with the same object. Note, this can include environmental enrichment |  |
|  | Playing with a conspecific | Engaging in active play with another elephant, including head to head sparring, trunk wrestling, mounting, chasing, and rolling on one another. Does not include behaviours observed following an antagonistic encounter or as part of courtship |  |
|  | Initiating play | An interaction between two elephants which leads to the initiation of play within 10 seconds |  |
|  | Floppy run | Running' with a loose, floppy gait, head is held low and trunk and ears are flapping around. Often accompanied by trumpeting. |  |
| **Proximity to others elephants** | Proximity | <3 or >3 elephant body length from another elephant | Day |

## * Behaviours only included in the Daytime ethogram are noted

## Appendix D in S1 Appendix: Final version of Behavioural Welfare Assessment Tool

**Section A**

**Live Observations – Qualitative Behavioural Assessment**

Elephant: ____________________________ Date: __________________________

Assessor: ______________________________________________

Number of years working with elephants ______

Please complete this short Qualitative Behaviour Assessment based on **1 minute** of live observation of this individual. For each term, draw **a single vertical mark** on the line between the two terms to indicate where along that scale you think the elephant falls.

***Content:*** *appears at ease, tranquil, seems satisfied*

| Not content |  | Content |
| --- | --- | --- |

***Relaxed:*** *Peaceful, seems free from tension.*

| Not relaxed |  | Relaxed |
| --- | --- | --- |

***Uncomfortable:*** *ill at ease without a clear context for any distress. Body, trunk, head postures un-relaxed and possibly changing frequently, appear fidgety.*

| Comfortable |  | Uncomfortable |
| --- | --- | --- |

***Agitated:*** *a state of uncertainty which can be accompanied by physical restlessness and over-reaction to stimuli e.g. trumpeting. Scanning environment in a tense and anxious fashion.*

| Not agitated |  | Agitated |
| --- | --- | --- |

***Tense:*** *body, head, trunk, held in a rigid fashion, un-relaxed reactions to stimuli.*

| Not tense |  | Tense |
| --- | --- | --- |

***Frustrated:*** *reacting to seeking a goal without success; can be violent (kicking, tusking, whacking with trunk, head pushing with body, head-on charge) towards others or objects or take the form of tossing objects about as a displacement activity. Angry body posture.*

| Not frustrated |  | Frustrated |
| --- | --- | --- |

***Wary:*** *sometimes nervous, paused reaction to some stimuli, unwilling to move in or out of an area, may be accompanied by listening and smelling. It is a slow and calm behaviour.*

| Not wary |  | Wary |
| --- | --- | --- |

***Playful:*** *engaged in a bout of object, locomotory or social play. Responds positively to solicitations for play.*

| Not playful |  | Playful |
| --- | --- | --- |

**Section B**

**Live Observations – Daytime activity**

Elephant: ____________________________ Date form completed: _____________

Assessor: ____________________________________

Number of years working with elephants ______

Please answer the following questions based on the live observations you have made over the last 3 days.

1. For the multiple choice questions, please choose the ONE best answer.
2. For the questions that have a line drawn between two descriptions at each end of the line (showing the range of possible answers), **draw a single vertical line** on the point along that line that you think best indicates where the elephant’s behaviour lies between those two points.

For example:

I hate driving ______________________________________ I love driving

**Section 1: Stereotypies**

- 1. During the last 3 days of observations this elephant has performed a stereotypy...

1. Always (few, if any, stereotypy-free intervals)
2. Very frequently (virtually every hour)
3. Frequently (daily, multiple times per day)
4. Occasionally (daily but infrequent)
5. Rarely (not daily)
6. Never
   1. If you have seen this elephant stereotype please give a breakdown of the stereotypies seen, their approximate frequency and the approximate time of day they occurred

| Description | Frequency (using above scale) | Time of day | Location |
| --- | --- | --- | --- |
|  |  |  |  |
|  |  |  |  |
|  |  |  |  |
|  |  |  |  |

- 1. Do the stereotypies this elephant performs ever interfere with their behaviour?

1. Stereotypies do not interrupt flow of behaviour
2. Stereotypies occasionally interrupt flow of behaviour
3. Stereotypies frequently interrupt flow of behaviour
4. Frequently interrupt flow of behaviour and occasionally disrupt intended action
5. Stereotypies frequently disrupt intended action
   1. Can the stereotypies this elephant performs be interrupted? Yes/No/Unknown

If yes, please describe what they are interrupted by:

**Section 2: Comfort Behaviour - Wallowing**

- 1. During the last 3 days of observations this elephant has wallowed…

1. More than once a day during my observations
2. Around once a day during my observations
3. Never during my observations

**Section 3: Feeding**

- 1. During the last 3 days of observations I have seen this elephant feeding...

1. Almost every time I looked at them
2. Most but not every time I have looked at them
3. Half or more of the times I have looked at them
4. Less than half the times I have looked at them
5. Around once a day during my observations
6. Less than daily during my observations
   1. During the last 3 days of observations I have seen this elephant...

| *Rarely forage and/or only feed at scheduled feed times* |  | *Forage for food all the time it is free to do so* |
| --- | --- | --- |

**Section 4: Activity**

4.1 During the last 3 days of observations I have seen this elephant...

| *Spend most of its day waiting for scheduled events* |  | *Engaging in activities completely independent of the scheduled events* |
| --- | --- | --- |

**Section 5: Social and Environmental Interactions**

5.1 During the last 3 days of observations I have seen this elephant **interacting** **with the environment** (investigating or interacting with things in the environment **other than food** with the trunk...

1. Almost every time I looked at them
2. Most but not every time I have looked at them
3. Half or more of the times I have looked at them
4. Less than half the times I have looked at them
5. Around once a day during my observations
6. Less than daily during my observations
7. Never during my observations

In a few words please describe with what they were interacting

5.2 During the last 3 days of observations I have seen this elephant...

| *Avoid other elephants every time it is free to do so* |  | *Spend time near or approach other elephants every time it is free to do so* |
| --- | --- | --- |

5.3 During the last 3 days of observations I have seen this elephant engaging in **affiliative behaviour** (any positive social interaction, e.g. touching another elephant in a non-aggressive manner) ...

1. Almost every time I looked at them
2. Most but not every time I have looked at them
3. Half or more of the times I have looked at them
4. Less than half the times I have looked at them
5. Around once a day during my observations
6. Less than daily during my observations
7. Never during my observations

In a few words please describe with whom they were interacting and how:

5.4 During the last 3 days of observations I have seen this elephant engaging in **agonistic behaviour** (any negative social interaction, behaving in a manner which causes harm or potential harm to conspecifics, e.g. displaces, displays, chases, bites) ...

1. Almost every time I looked at them
2. Most but not every time I have looked at them
3. Half or more of the times I have looked at them
4. Less than half the times I have looked at them
5. Around once a day during my observations
6. Less than daily during my observations
7. Never during my observations

In a few words please describe with whom they were interacting and how:

5.5 During the last 3 days of observations I have seen this elephant **playing with conspecifics** (engaging in active play with another elephant, including head to head sparring, trunk wrestling, mounting, chasing, and rolling on one another. Does not include behaviours observed following an antagonistic encounter or as part of courtship) ...

1. More than once a day during my observations
2. Around once a day during my observations
3. Never during my observations

In a few words please describe with whom they were interacting and how:

**Section 6**

Please provide details of any other observations you have made of this animal in the last 3 days which you believe are of importance

**Section 7**

**Please answer the following questions based on your general experience from working with this elephant:**

7.1 When was the last time you saw this elephant come across a new or unexpected situation? What was the situation and what was their reaction?

7.2 Please describe in the table below any vocalisations this individual has made and the contexts in which they occurred

| Vocalisation – Rumble, Trumpet or Other (please provide short description) | Context |
| --- | --- |
|  |  |
|  |  |
|  |  |

**Section 8**

**Overall, at the current point in time...**

Please place a single vertical mark along the line to indicate what you feel is the appropriate location for each aspect of health and welfare for this individual.

**8.1 Mental health**

*Worst imaginable*

*Best imaginable for any elephant anywhere*

**8.2 Physical health**

*Worst imaginable*

*Best imaginable for any elephant anywhere*

**8.3 Overall Welfare**

*Worst imaginable*

*Best imaginable for any elephant anywhere*

**Comments:** Please provide any other comments relating to this this elephant, or your assessment of it.

**Section B Crib Sheet: Elephant Behaviour Notes**

| Elephant Name:  Dates of Observation: | | | | | | | | | | |
| --- | --- | --- | --- | --- | --- | --- | --- | --- | --- | --- |
| **Behaviours observed** | | | | | | | | | | |
| **Day** | **Time Block** | Stereotyping | Wallowing | Feeding | Foraging | Environmental Interaction | Affiliative | Agonistic | Affiliative: Conspecific play | Anticipating |
| **One** |  |  |  |  |  |  |  |  |  |  |
|  | 9:00 – 11:00 |  |  |  |  |  |  |  |  |  |
|  | 11:00 – 13:00 |  |  |  |  |  |  |  |  |  |
|  | 13:00 – 15:00 |  |  |  |  |  |  |  |  |  |
|  | 15:00 – 17:00 |  |  |  |  |  |  |  |  |  |
| **Two** |  |  |  |  |  |  |  |  |  |  |
|  | 9:00 – 11:00 |  |  |  |  |  |  |  |  |  |
|  | 11:00 – 13:00 |  |  |  |  |  |  |  |  |  |
|  | 13:00 – 15:00 |  |  |  |  |  |  |  |  |  |
|  | 15:00 – 17:00 |  |  |  |  |  |  |  |  |  |
| **Three** |  |  |  |  |  |  |  |  |  |  |
|  | 9:00 – 11:00 |  |  |  |  |  |  |  |  |  |
|  | 11:00 – 13:00 |  |  |  |  |  |  |  |  |  |
|  | 13:00 – 15:00 |  |  |  |  |  |  |  |  |  |
|  | 15:00 – 17:00 |  |  |  |  |  |  |  |  |  |
| Comments: | | | | | | | | | | |

**Section C: Datasheet for overnight behaviour**

Name of elephant: Date: Assessor:

| **Time** | **Behaviours Observed** | | | | | | | |
| --- | --- | --- | --- | --- | --- | --- | --- | --- |
|  | **Feeding** | **Lying Down:**  **ALONE or WITH OTHERS**  **(if with others, names of others)** | **Stereotypy** | **Comfort** | **Interaction with Environment** | **Social/**  **Affiliative** | **Other (describe)** | **Out of view** |
| 21:00 |  |  |  |  |  |  |  |  |
| 21:30 |  |  |  |  |  |  |  |  |
| 22:00 |  |  |  |  |  |  |  |  |
| 22:30 |  |  |  |  |  |  |  |  |
| 23:00 |  |  |  |  |  |  |  |  |
| 23:30 |  |  |  |  |  |  |  |  |
| 00:00 |  |  |  |  |  |  |  |  |
| 00:30 |  |  |  |  |  |  |  |  |
| 01:00 |  |  |  |  |  |  |  |  |
| 01:30 |  |  |  |  |  |  |  |  |
| 02:00 |  |  |  |  |  |  |  |  |
| 02:30 |  |  |  |  |  |  |  |  |
| 03:00 |  |  |  |  |  |  |  |  |
| 03:30 |  |  |  |  |  |  |  |  |
| 04:00 |  |  |  |  |  |  |  |  |
| 04:30 |  |  |  |  |  |  |  |  |
| 05:00 |  |  |  |  |  |  |  |  |
| 05:30 |  |  |  |  |  |  |  |  |
| 06:00 |  |  |  |  |  |  |  |  |
| 06:30 |  |  |  |  |  |  |  |  |
| 07:00 |  |  |  |  |  |  |  |  |
| 07:30 |  |  |  |  |  |  |  |  |
| 08:00 |  |  |  |  |  |  |  |  |
| 08:30 |  |  |  |  |  |  |  |  |
| 09:00 |  |  |  |  |  |  |  |  |

Incidents of aggression (agonistic behaviour)

| Time | Behaviour seen | Circumstances | Aggressor(s) | Recipient(s) |
| --- | --- | --- | --- | --- |
|  |  |  |  |  |
|  |  |  |  |  |
|  |  |  |  |  |

**Comments:**

**Definition of terms for behaviour assessment**

The behavioural terms used in Sections B & C of the Behavioural Welfare Assessment Tool are defined below. The category of each behaviour is given, along with the term used to describe that behaviour, and the definition of each term.

| **Category** | **Behaviour** | **Description** |
| --- | --- | --- |
| **Sleep/Rest** | **Lying rest** | Lateral recumbency, no other behaviours are being performed |
| **Anticipatory** | **Anticipating** | Standing alert, often near (within two elephant body lengths) of gates or enclosure bars prior to an event (e.g. feeding, moving inside, etc.) |
| **Stereotyping** | | Repetitive behaviour with no obvious purpose. May include but is not limited to the following**:** |
|  | **Head bobbing** | Repetitive vertical movement of head |
|  | **Swaying/**  **Weaving** | Side to side or back and forth repetitive swaying of the body |
|  | **Trunk tossing** | Vigorous swaying of trunk from side to side |
|  | **Head pressing** | Pressing head up against an object with no oblivious purpose |
|  | **Pacing** | Walking repeatedly along the same route in an unvarying, repetitive pattern |
|  | **Leg swing** | Standing still repeatedly swinging one front leg back and forth |
|  | **Foot lifting** | Standing still repeatedly lifting one foot in the air |
|  | **Rocking** | Rocking back and forth transferring weight from hind to front legs |
|  | **Tusk banging** | Repetitive banging or rubbing of the tusks on objects (e.g. enclosure bars or logs) |
|  | **Bar biting** | Chewing or gnawing on enclosure bars |
| **Comfort** | | Any self-maintenance or grooming behaviour. May include but is not limited to: |
|  | **Wallowing** | Lying down and rolling in mud |
|  | **Rubbing** | Rubbing the body against an object |
|  | **Rolling** | Lying down and rolling in dirt or sand |
|  | **Scratching** | Scratching the body with trunk or foot - this can include using the trunk to scratch/feel gently around the skin, eye or ear |
|  | **Scratching with tool** | Scratching the body with a tool, e.g. branch or stick |
|  | **Throwing straw on self** | Throwing soft objects such as grass or straw onto the body using the trunk |
|  | **Dust bath** | Spraying mud, dust or sand on the body |
|  | **Water bath** | Spraying water on the body |
|  | **Body slap** | Hitting of own body with tail or trunk, appears to be a means of removing an insect or other irritant |
| **Social/affiliative** | | **Any positive or neutral interaction with another elephant. May include but is not limited to:** |
|  | **Leaning** | Leaning on another elephant |
|  | **Standing** | Standing underneath or standing above another elephant |
|  | **Trunk-mouth** | Putting the trunk in the mouth of another elephant |
|  | **Touching** | Touching another elephant with the trunk in a non-aggressive manner |
|  | **Tail** | Holding the tail of another elephant with the trunk or underneath a leg |
|  | **Trunk-trunk** | Intertwining of trunks between two elephants |
|  | **Approach** | Moving in a non-aggressive manner, within one body length of another elephant |
|  | **Climb** | Placing at least one foot on top of another elephant - usually one that is lying down |
|  | **Offer food** | One elephant pushes a pile of food towards another elephant, looks like an offering of the resource |
|  | **Trunk lifting** | Trunk is outstretched and raised towards an approaching individual |
|  | **Sitting on an elephant** | Sitting in a crouched position on top of another elephant which is in lying rest |
|  | **Rubbing elephant** | Rubbing the body against another elephant |
|  | **Playing with an elephant** | Engaging in active play with another elephant, including head to head sparring, trunk wrestling, mounting, chasing, and rolling on one another. Does not include behaviours observed following an antagonistic encounter or as part of courtship |
| **Interaction with environment** | | **Investigating or interacting with things in the environment (other than food). May include but is not limited to:** |
|  | **Play with an object** | Throwing or kicking debris or an object around in a playful interaction |
|  | **Object manipulation** | Examination or manipulation of an object with the trunk and/or foot |
|  | **Environmental investigation** | Investigating things in the environment (other than food) with the trunk - looks like the individual may be 'sniffing' at the ground or objects |
| **Eating/Foraging** | **Eating** | Manipulation (including breaking up of food stuffs using the trunk or foot) and consumption of food |
|  | **Foraging** | The process of searching for and collecting food stuffs using the trunk and/or foot - may include kicking up grasses, shaking the food in the trunk or beating against the leg |
| **Agonistic behaviour** | | **Any negative social behaviour** |
|  | **Tusking** | Poking or jabbing at another elephant with the tusk |
|  | **Charge** | Move towards another elephant with the head held high, pace usually quickens as individual gets closer to the target elephant, can lead to pursuit of another elephant |
|  | **Bite** | Biting of the body, trunk or tail of another elephant |
|  | **Kick** | Strike out or hit an elephant or object with a foot in a seemingly aggressive manner - note object may include enclosure bars or kicking of sand towards another elephant |
|  | **Strike** | Hitting another elephant with the trunk or tail |
|  | **Push** | One elephant forces or pushes against the body (usually the rump) of another elephant, resulting in the elephant that is being pushed moving at least two steps |
|  | **Stand off** | Two elephants standing facing in opposite directions with foreheads pushing against each other |
|  | **Smack** | Hitting the trunk on the floor in an aggressive manner, may be accompanied by a 'snort' |
|  | **Food stealing** | Taking food from another elephant |
